# Frontier and hot topics in the application of hydrogel in the biomedical field: a bibliometric analysis based on CiteSpace

**DOI:** 10.1186/s13036-024-00435-2

**Published:** 2024-07-23

**Authors:** Weiming Sun, Wendi Wu, Xiangli Dong, Guohua Yu

**Affiliations:** 1 Department of Rehabilitation Medicine, The First Affiliated Hospital, Jiangxi Medical College, Nanchang University, Nanchang, 330006 China; 2 Postdoctoral Innovation Practice Base, The First Affiliated Hospital, Jiangxi Medical College, Nanchang University, Nanchang, 330006 China; 3 Queen Mary School, Jiangxi Medical College, Nanchang University, Nanchang, 330031 China; 4 Department of Psychosomatic Medicine, The Second Affiliated Hospital, Jiangxi Medical College, Nanchang University, Nanchang, 330006 China

**Keywords:** Hydrogel, Biomedical, Bibliometrics, Citespace, Hot spot, Wound healing, Controlled release, Drug delivery, Tissue engineering

## Abstract

Hydrogels are formed of crosslinked polymer chains arranged in three-dimensional (3D) networks. These chains have good water-containing capacity and are soft and malleable. Hydrogels have good biocompatibility due to their significant water content, flexible structure, and numerous holes. These characteristics make them analogous to biological tissues. Despite the publication of 8700 literature related to hydrogel biomedical applications in the past 52 years (1973 ~ 2024), studies on the use of hydrogels in biomedicine are few. To gain a comprehensive understanding of their current development status, research trends, and prospects in the biomedical application field, it is imperative to conduct a thorough retrospective analysis. In this study, we employ bibliometric analysis and CiteSpace software to quantitatively and visually analyze articles published in this field. Firstly, we provide a quantitative analysis of authorship and institutional publications over the past 52 years to elucidate the fundamental development status regarding hydrogel biomedical applications. Secondly, we did visual studies on terms that are high-frequency, explosive, keyword clustering, and so on, to understand the directionality and evolution of the main research hotspots during each period. Notably, our findings emphasize that fabricating hydrogels into wound healing-promoting dressings emerges as a prominent hotspot within the application field. We anticipate that this paper will inspire researchers with novel ideas for advancing hydrogel applications in biomedicine.

## Introduction

Crosslinked polymer chains create three-dimensional (3D) networks known as hydrogels, which are highly capable of absorbing water and have pliable characteristics. [1]. Owing to their large water content, flexible structure, and multipoles, hydrogels have outstanding biocompatibility and mimic live tissues quite a bit. Consequently, hydrogels have gained increasing prominence in the field of biomedical research [2]. Depending on their origin, hydrogels can be classified as natural, synthetic, and semi-synthetic hydrogels. Natural hydrogels encompass agarose, alginate, chitosan, collagen, elastin-like peptides, fibrin, gelatin, hyaluronic acid, methylcellulose, and so on [3]. These hydrogels consistently show properties of bioactivity, biocompatibility, and biodegradability; nevertheless, their mechanical strength and stability are somewhat restricted [4]. Some natural hydrogels may induce allergies and pose immune risks when directly applied to the human body [5]. Synthetic hydrogels consist of multiple monomers that possess specific biocompatibility and robust mechanical potency [6]. Chemically altered natural hydrogels or blends of natural and synthetic hydrogels are known as semi-synthetic hydrogels [7, 8]. These hydrogels modify their properties chemically while retaining the bioactive qualities of the natural ones [9].

Hydrogels have been discovered and extensively investigated by scientists for several decades. The first hydrogels used for biomaterials were created in 1960 thanks to the groundbreaking work of Wichterle and Lim. Specifically, a synthetic poly-2-hydroxyethyl methacrylate (PHEMA) hydrogel was used as a filling agent following enucleation and contact lens creation [10]. Initially, from 1970 to 1990, hydrogels found applications primarily on the body surface, such as eyes or open wounds [11]. Not only that hydrogels have also been used in functional wound dressings in recent years. The physical characteristics of hydrogels make it possible for a barrier to develop against outside influences and for excess exudates to be absorbed by water, thereby creating a moist environment that fosters wound healing. Furthermore, hydrogels can be utilized to effectively fill irregularly shaped wounds, aiding in their reparative process [12]. Moreover, hydrogels also exhibit highly advantageous biological activities including antibacterial and anti-inflammatory properties, as well as promoting blood coagulation and regeneration [13]. Since 1990, there has been a gradual expansion of utilization in other fields, such as tissue engineering and drug delivery systems [14]. Tissue engineering involves the development of a scaffold that mimics the body’s extracellular matrix to address tissue failure and impaired self-repair. Because of their strong mechanical characteristics, biocompatibility, biodegradability, and similarity to the extracellular matrix in vivo, hydrogels are widely used in tissue engineering. For instance, hydrogel scaffolds can be utilized for regenerating tissues such as nerves, cardiac muscle, cartilage, and bone [15]. Hydrogels also demonstrate exceptional responsiveness to stimuli and possess the ability to modify their properties by the surrounding environment, thereby facilitating precise drug release and safeguarding labile pharmaceuticals against degradation. Consequently, hydrogels have emerged as highly efficacious platforms for drug delivery systems [16]. With recent advancements in understanding the in situ gelation process following infection and stimulus reactivity of hydrogels, these versatile biomaterials are no longer confined solely to surface environments but can be designed for implantation, injection, or spray administration within various organs and tissues [17, 18].

Bibliometrics, as a subfield of informatics, employs quantitative analyses of scientific literature to discern emerging trends. It serves as an invaluable tool for researchers to objectively investigate the current state and progression of their discipline, while also identifying pioneering advancements. By utilizing bibliometric analysis software, researchers can augment the quality of their literature reviews by constructing knowledge maps [19]. Among many kinds of analysis software, CiteSpace is a popular tool in information research since it is a web-based Java program intended for data analysis and visualization. It employs metrology, co-occurrence analysis, cluster analysis, and pathway exploration to create visual maps that uncover network structures, enabling researchers to pinpoint study areas, annotate keywords, spot developing patterns (turning points), and recognize abrupt shifts over time [20]. To help researchers have a better understanding of future research directions, this study used CiteSpace to examine the hot areas and research frontiers of hydrogel biomedical applications.

## Data sources and analysis methods

### Data source

Web of Science is a comprehensive academic information resource, recognized as the world’s largest, encompassing diverse disciplines. It encompasses an extensive collection of over 8,700 core academic journals that wield substantial influence across various research fields such as natural science, engineering technology, and biomedicine. By harnessing the robust retrieval capabilities offered by the Web of Science platform, researchers can effortlessly and expeditiously access invaluable scientific research information to acquire a profound understanding of specific disciplines and subjects. We used the Web of Science Core Collection database to strengthen the representativeness and accessibility of the data. We used “#1 Topic Sentence = hydrogel?, #2 Topic Sentence = medic* or biomedic* or “medical application” or “biomedical application” or cancer or tumor or disease or diagnos* or therap* or physiolog* or biosens* or bioassay* or antibacteri* or antimicrob*, #3 = #2 and #3” as the search sentence to search literatures in SCI-EXPANDED index with the time span of 1973 ~ 2024. There are a total of 2862 records, 2788 article, 10 book chapter, 25 early access and 39 proceedings paper.

### Analysis method

The 6.3.R1W version CiteSpace program is the primary instrument utilized in this article to do an extensive literature analysis of the chosen works. When it comes to data processing, the source chooses several items, including the title, abstract, supplemental keywords and author keywords. The selection period spans from 1973 to 2024, and the time slice is one year. For the node, choose Author, Institution, Country, Keyword, Category, Reference, Cited Author and Cited Journal. For the threshold, choose Top = 50. Leave the other values as they are. Using the theoretical framework of cluster analysis, we conducted a retrospective study and summary of the literatures in the field of hydrogel biomedical application during the previous 52 years, putting up addressing network techniques (PF-NET, Pathfinder Network Scaling). We use the program to create a knowledge map, co-occurrence maps to look into research trends across time, and temporal views to look into the links that arise between these research hotspots.

## Results

### Quantitative analysis of basic information

#### Annual publication trend

**Fig. 1:**
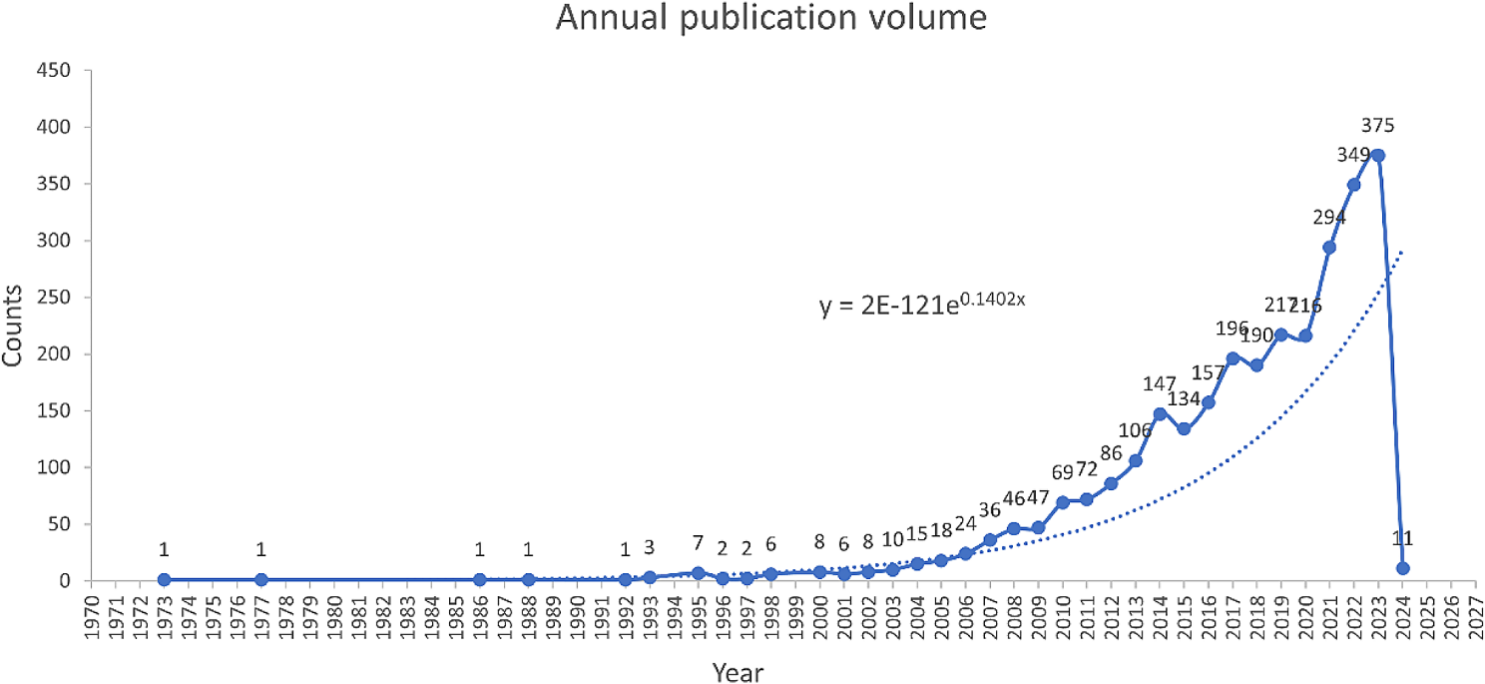
Annual publication output in the hydrogel biomedical application field from 1973 to 2024

The number of publications published in the hydrogel biomedical application sector between 1973 and 2024 was recorded in this study. The yearly publication volume data in this discipline indicate an increasing number of publications annually, as Fig. 1 illustrates. As early as 1973, researchers directed their attention towards this field. Subsequently, starting in 2003, there was a steady rise in the number of articles published in this field. However, it was after 2009 that a significant surge in publications occurred, and post-2015 witnessed an exponential rise, indicating a notable shift in researchers’ focus toward this area of study. Remarkably, 294 papers were published in this discipline in 2021, which is an astounding increase of 78 articles over the previous year and a noteworthy rise. Looking ahead to the first year of 2024 alone, already eleven articles have been published thus far, demonstrating its sustained activity. We predict that in 2024, there will be more published papers than 400, which is a demonstration of the researchers’ persistent concern in pursuing and discovering the research potential in this discipline, especially considering the recent quick expansion.

#### Analysis of published journals

In the literature we selected, we identified the top 10 journals (Table 1) based on their prolific publication records.

Acta Biomaterialia topped the list with 302 articles, Biomaterials came in second with 298 publications, and Frontiers in Bioengineering and Biotechnology came in tenth with 68 publications. In addition to having the second-largest number of articles, biomaterials had the greatest impact factor (14 in 2022) and the highest CiteScore (23.4 in 2022). Biomaterials’ h-index was 418. The h-index is a quantitative metric that assesses the scholarly output of a journal by quantifying the number of highly cited papers and their citation impact. Journals with higher h-indices are generally considered to possess greater academic influence. Biomaterials is a globally recognized journal that focuses on the scientific exploration and clinical applications of biomaterials. Its primary objective is to comprehensively address the multifaceted aspects associated with the utilization of biomaterials in clinical practice, encompassing original research papers, authoritative reviews, and opinion papers. Among the top ten journals, half of them were published by the Elsevier publishing unit. It is evident that Elsevier exhibited a profound interest in this field and in the future, there will be opportunities for researchers who hammered at this domain to consider submitting their articles to the numerous journals of Elsevier.

**Fig. 2:**
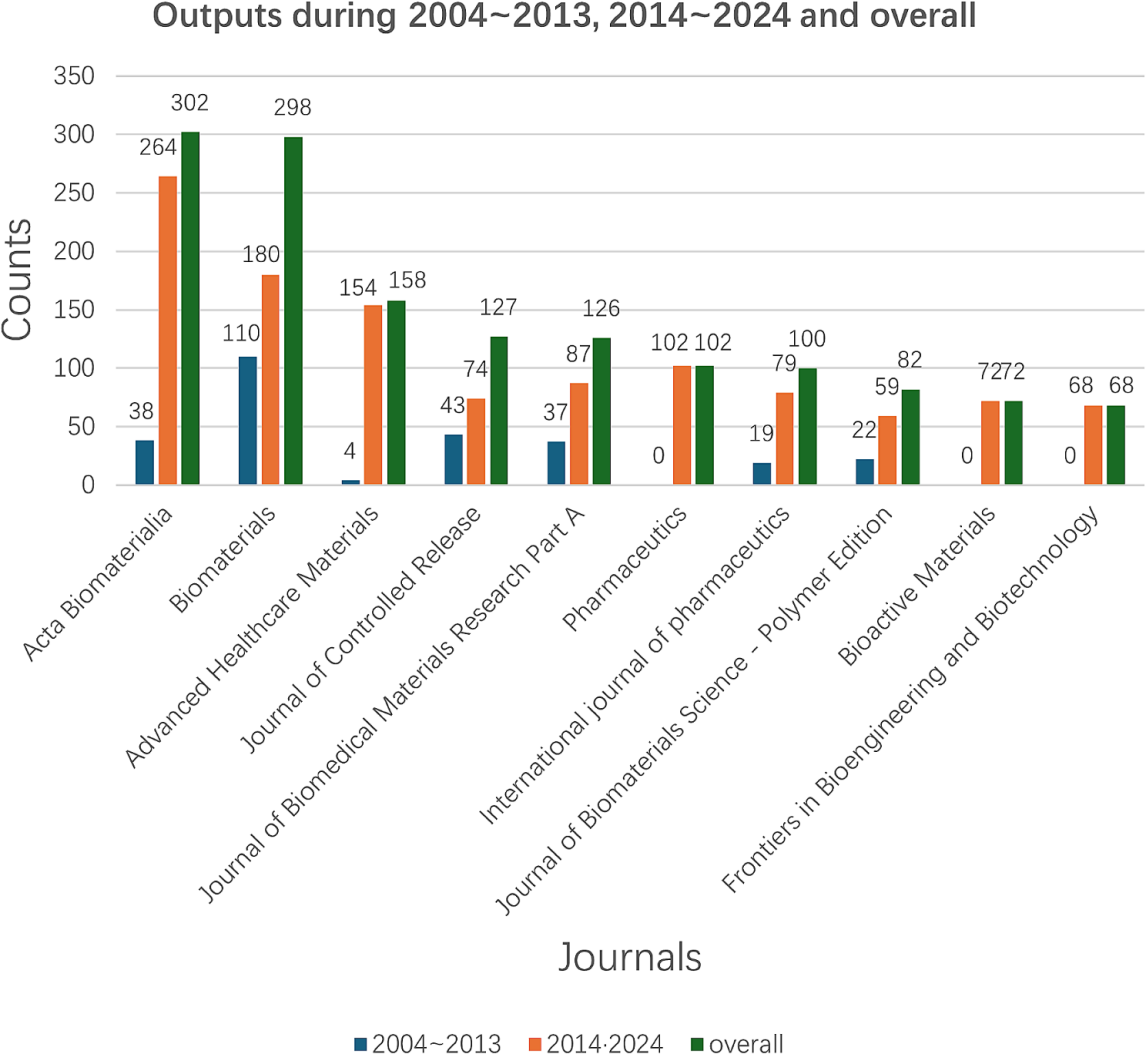
Publication outputs of the Top 10 journals in the hydrogel biomedical application field during 2004 ~ 2013, 2014 ~ 2024 and overall

**Fig. 3:**
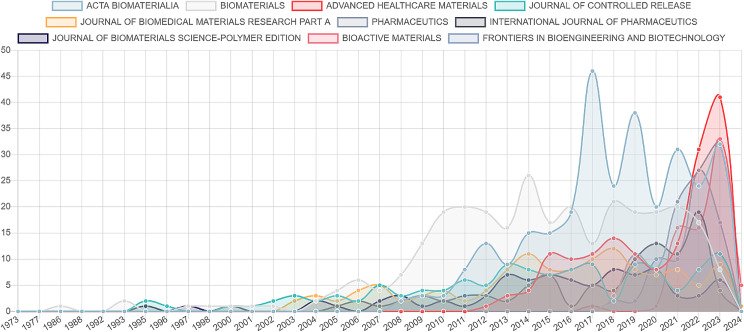
Top 10 publication output journals in the hydrogel biomedical application field in the past 52 years

According to Fig. 1, most works have been published in the past two decades, but there are significant differences in output between these periods. We analyzed the number of articles published by the top 10 journals during 2004–2013 and 2014–2024. Figure 2 illustrates the output of these top 10 journals across 2004–2013, 2014–2024, and overall. Figure 3 reveals that all 10 journals have published significantly more articles in the recent decade (2014–2024), indicating that the application of hydrogels in biomedicine has become a hot research field. Among these journals, Pharmaceutics, Bioactive Materials, and Frontiers in Bioengineering and Biotechnology are particularly noteworthy, as they only began publishing articles in this field after 2017.

**Table 1:** Top 10 journals with publication output from 1973 to 2024

Number	Journal	Counts	CiteScore	IF (2022)	Publisher
1	Acta Biomaterialia	302	17	9.7	Elsevier
2	Biomaterials	298	23.4	14	Elsevier
3	Advanced Healthcare Materials	158	15.5	10	Wiley
4	Journal of Controlled Release	127	17.1	10.8	Elsevier
5	Journal of Biomedical Materials Research Part A	126	10	4.9	Wiley
6	Pharmaceutics	102	6.9	5.4	MDPI
7	International journal of pharmaceutics	100	10.5	5.8	Elsevier
8	Journal of Biomaterials Science - Polymer Edition	82	6.4	3.6	Taylor & Fancis group
9	Bioactive Materials	72	19.7	18.9	Elsevier
10	Frontiers in Bioengineering and Biotechnology	68	6.7	5.7	Frontiers

### Cooperative relationship network

#### Country cooperation network

**Fig. 4:**
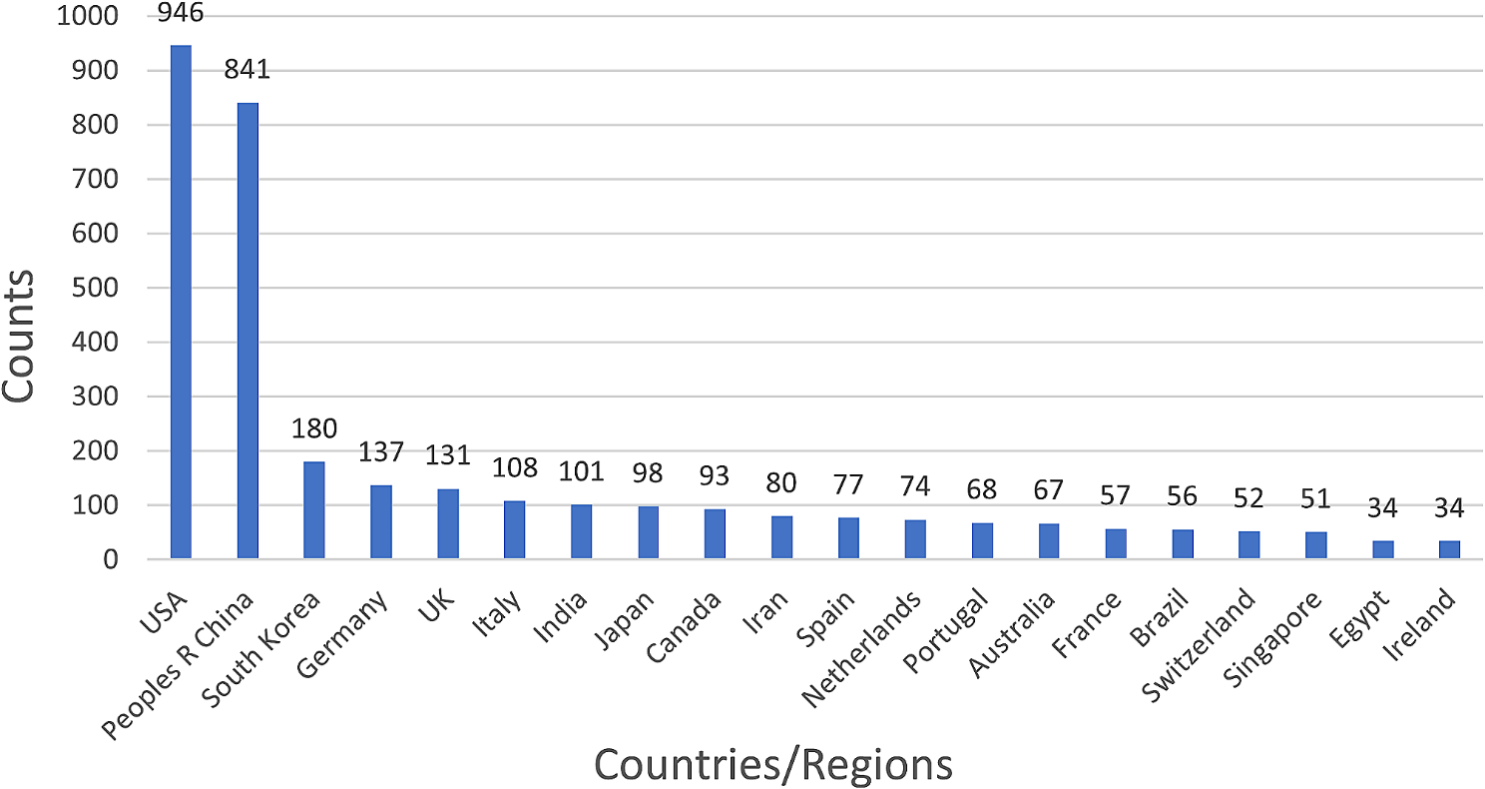
Top 20 countries in the field of hydrogel biomedical application

We carried out a thorough statistical analysis of the publications from different nations (regions) and organizations. Through our investigation, we successfully identified nations and institutions that exhibited a substantial volume of published papers while exerting significant influence within hydrogel biomedical application field. Furthermore, we meticulously examined the collaborative relationships established among these entities. Articles regarding hydrogel biomedical appliocation were published by 2706 institutions in 79 countries and regions between 1973 and 2024. The top 10 nations were as follows: USA, China, South Korea, Germany, UK, Italy, India, Japan, Canada, and Iran. The top 20 institutions were displayed in Fig. 4. The statistics show that, with 946 papers published and 33.05% papers of all, the USA had the greatest number of articles published. China was not far behind, 791 articles accounts for 27.61% of the entire counts of articles. This indicates that USA and China had leader position in the field of hydrogel biomedical application.

**Fig. 5:**
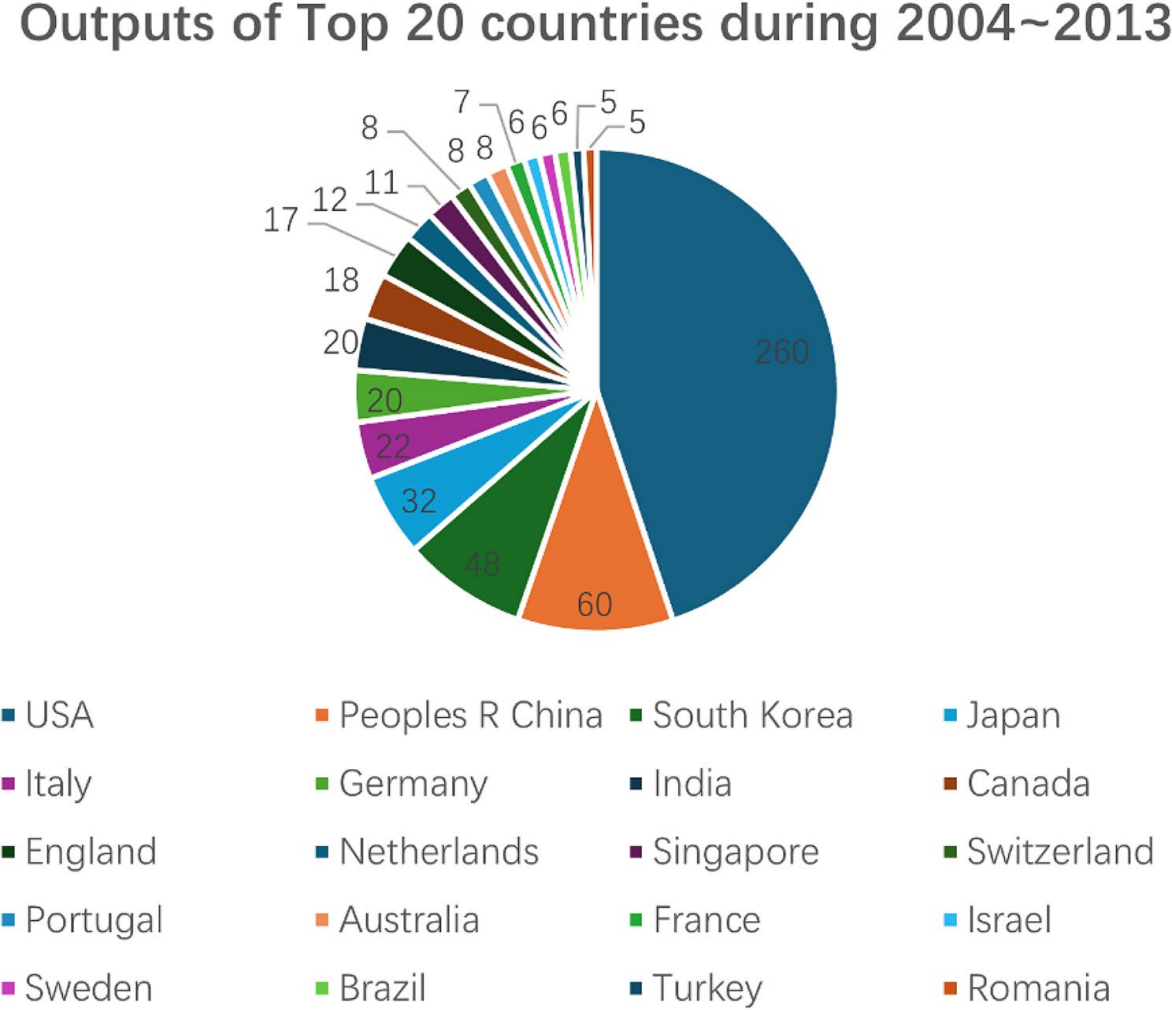
Top 20 countries in the field of hydrogel biomedical application during 2004 ~ 2013

**Fig. 6:**
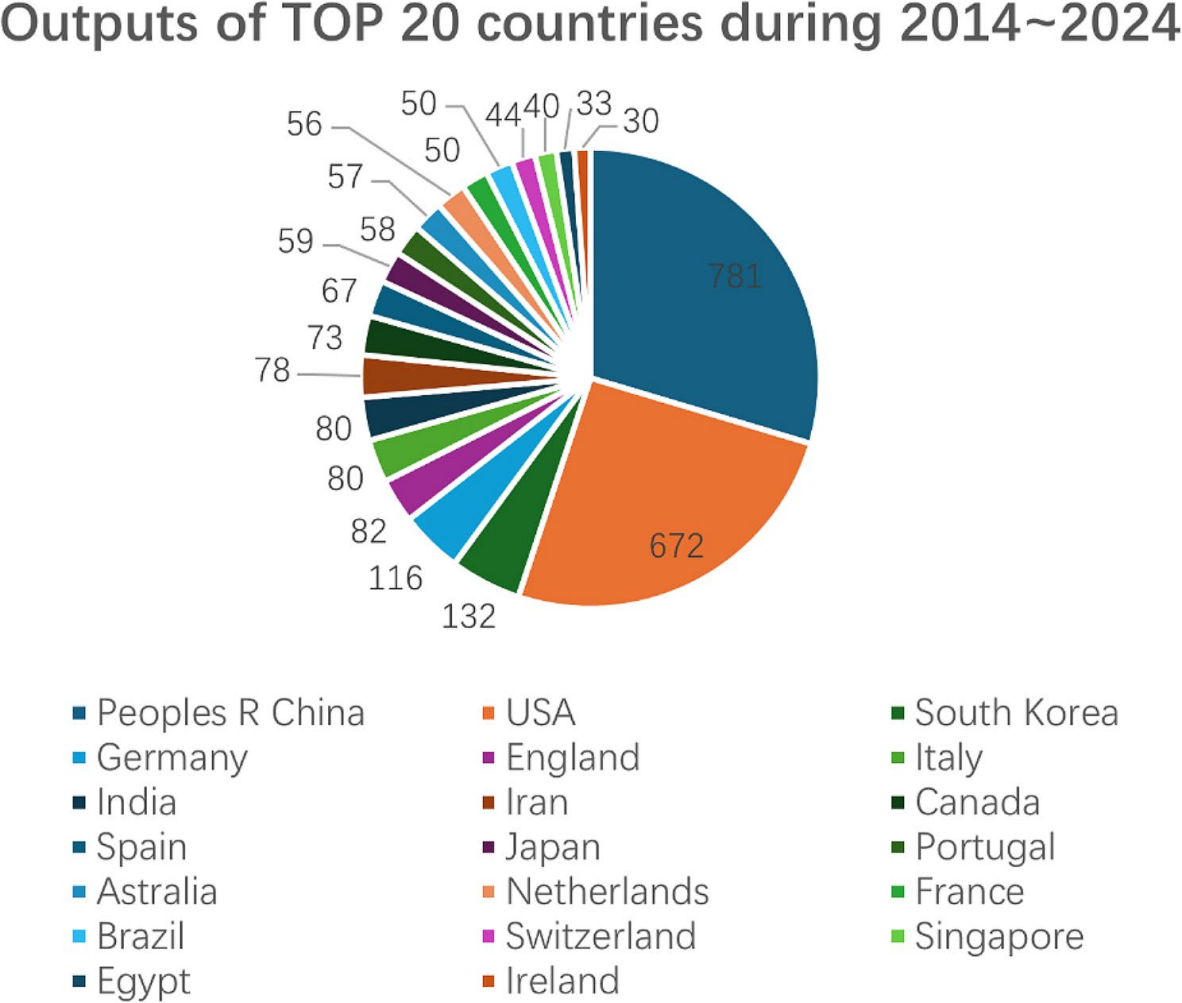
Top 20 countries in the field of hydrogel biomedical application during 2014 ~ 2024

By further analyzing the outputs of the top 20 countries during 2004–2013 and 2014–2024, we present the results in Figs. 5 and 6. Notably, the United States, China, and South Korea have consistently ranked as the top three countries in hydrogel research over the past two decades. This trend aligns with their positions in terms of total publication output over the last 52 years. However, a significant shift has occurred in recent years, particularly within the last decade, where Chinese authors have experienced a remarkable surge in article publications, establishing China as the leading country in terms of sheer volume. Notably, all three countries have seen a substantial increase in published documents in the past ten years compared to the previous decade. Consequently, it is evident that hydrogel applications in biomedicine have become a prominent subject during this period.

**Fig. 7:**
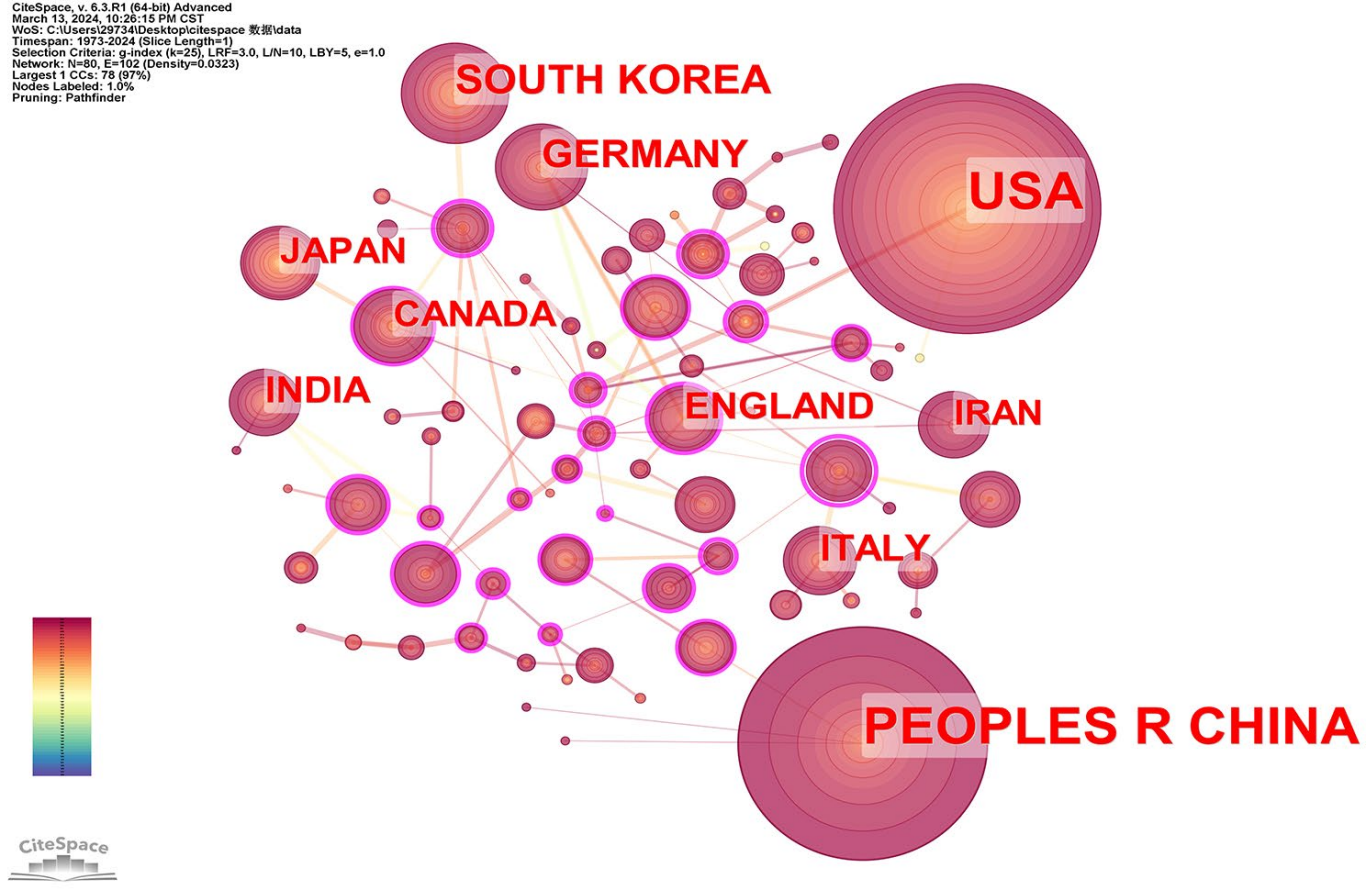
Network diagram of national cooperation in the field of hydrogel biomedical application

The cooperative relationship between countries was analyzed using the CiteSpace software (Fig. 7). The size of the node corresponds to the citation number of published papers, while the strength of cooperation is indicated by connecting lines between nodes. From nodes’ size, we can have strong evidence which can support the conclusion that the USA and China have incredible influence in this field. As a measure of how strongly a node is connected to other nodes in the network, centrality is an important standpoint in cooperative networks. A high degree of centrality indicates that important nodes have a big impact on the connections within the network. The nodes with purple circle contour have strong centrality. Among these countries, England and Canada held the prominent central position, this shows they both maintained close collaboration. Additionally, India also established robust collaborative partnerships. It is noteworthy that despite the USA and China ranked first and second in terms of paper publication and citation counts, their centrality scores were relatively low, indicating limited international collaboration. This observation may be attributed to their advanced research capabilities.

#### Institutional Cooperation Network

**Fig. 8:**
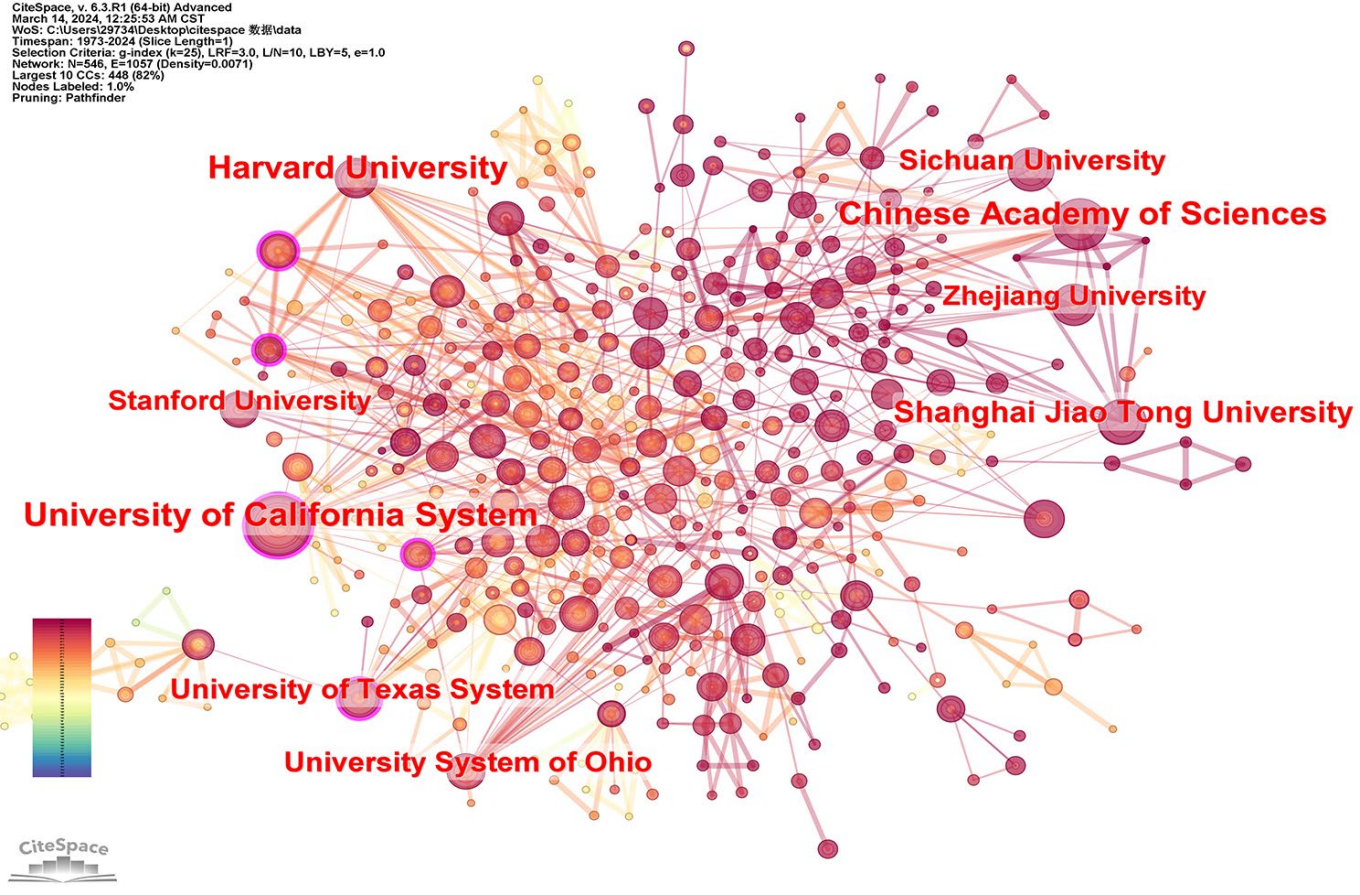
Institutional cooperation network in the field of hydrogel biomedical application

According to the statistical results of publication numbers and CiteSpace analysis, it is evident that the top 2 institutions in terms of publications are Chinese, thereby emphasizing China’s leadership position in this field (Table 2; Fig. 8). The Chinese Academy of Sciences secured the first rank with a total of 51 publications. Among the top ten institutions in terms of publication amount, four are Chinese institutions. Additionally, four other institutions in the top 10 are from the United States. Hence, both China and the United States wield crucial influence in this field. Although Chinese institutions published marginally more articles than their American counterparts, the disparity was not statistically significant. In the future, Chinese researchers must continue to pursue further studies in this domain to sustain their global leadership position.

**Table 2:** Top 10 institutions with the counts of publications from 1973 to 2024

Numbers	Affiliation	Counts	Countries/Regions
1	Chinese Academy of Sciences	51	PEOPLES R CHINA
2	Shanghai Jiao Tong University	50	PEOPLES R CHINA
3	Harvard University	47	USA
4	Sichuan University	45	PEOPLES R CHINA
5	Zhejiang University	41	PEOPLES R CHINA
6	University of Michigan	40	USA
7	Stanford University	39	USA
8	Kyoto University	36	Japan
9	MIT	36	USA
10	Universidade do Minho	36	The Portuguese Republic

In terms of inter-institutional collaboration, we used CiteSpace to identify the top ten institutions based on their citation counts, the results are shown in Table 3. The table illustrates that six out of the ten institutions are affiliated with the United States, they are the University of California System, Harvard University, University of Texas System, University System of Ohio, Stanford University, and Massachusetts Institute of Technology (MIT). The remaining four originate from China, they are the Chinese Academy of Sciences, Shanghai Jiao Tong University, Sichuan University, and Zhejiang University. Both American and Chinese researchers held prominent positions in this field, as their scholarly articles have garnered substantial citations from researchers worldwide.

**Table 3:** Top 10 institutions with the counts of citations from 1973 to 2024

Numbers	Affiliation	Counts	Countries/Regions
1	University of California System	85	USA
2	Harvard University	71	USA
3	Chinese Academy of Sciences	70	Peoples R China
4	Shanghai Jiao Tong University	51	Peoples R China
5	University of Texas System	42	USA
6	Sichuan University	41	Peoples R China
7	University System of Ohio	39	USA
8	Zhejiang University	36	PEOPLES R CHINA
9	Stanford University	35	USA
10	MIT	33	USA

As previously mentioned, centrality serves as an indicator of the nodes’ influence within the network. We utilized CiteSpace to generate a ranked list of the top ten nodes based on their centralities. The results are shown in Table 4. The majority of institutions exhibiting a high degree of centrality are located in the United States, with only Fudan University from China ranking among the top 10. It is evident that despite Chinese institutions having a substantial number of publications and citations, their impact on inter-agency collaboration remains limited. In the future, China’s numerous research institutions must engage in sincere collaborations aimed at achieving mutual benefits and gradually enhancing their academic influence.

**Table 4:** Top 10 institutions with the centralities

Numbers	Affiliation	Centrality	Countries/Regions
1	MIT	0.13	USA
2	University of Texas System	0.13	USA
3	Cornell University	0.12	USA
4	University of California System	0.11	USA
5	Brigham & Women’s Hospital	0.11	USA
6	Fudan University	0.1	PEOPLE R CHINA
7	University System of Ohio	0.09	USA
8	Harvard University	0.09	USA
9	Indian Institute of Technology System	0.09	India
10	Georgia Institute of Technology	0.08	USA

#### Author cooperation network

The analysis of the author’s collaboration network revealed that there were a total number of 13,643 researchers who contributed to the investigation of this network in hydrogel biomedical applications. In addition, we can see the rank of the top 20 writers in Table 5 60% of the authors in the table are from the USA, 15% of the authors are from China and 15% of the authors are from Korea. The aforementioned observation provides additional substantiation for the prominent position held by scholars hailing from both the United States. The top 20 scholars all published more than 10 articles. The first one is Professor Burdick Jason A. He is a professor from the University of Colorado Boulder, who published 22 articles in this field. His research fields encompass the development of injectable hydrogels for disease therapy, bioprinting tissue models for therapeutic drug screening, and fabricating fibrous scaffolds for tissue repair.

**Fig. 9:**
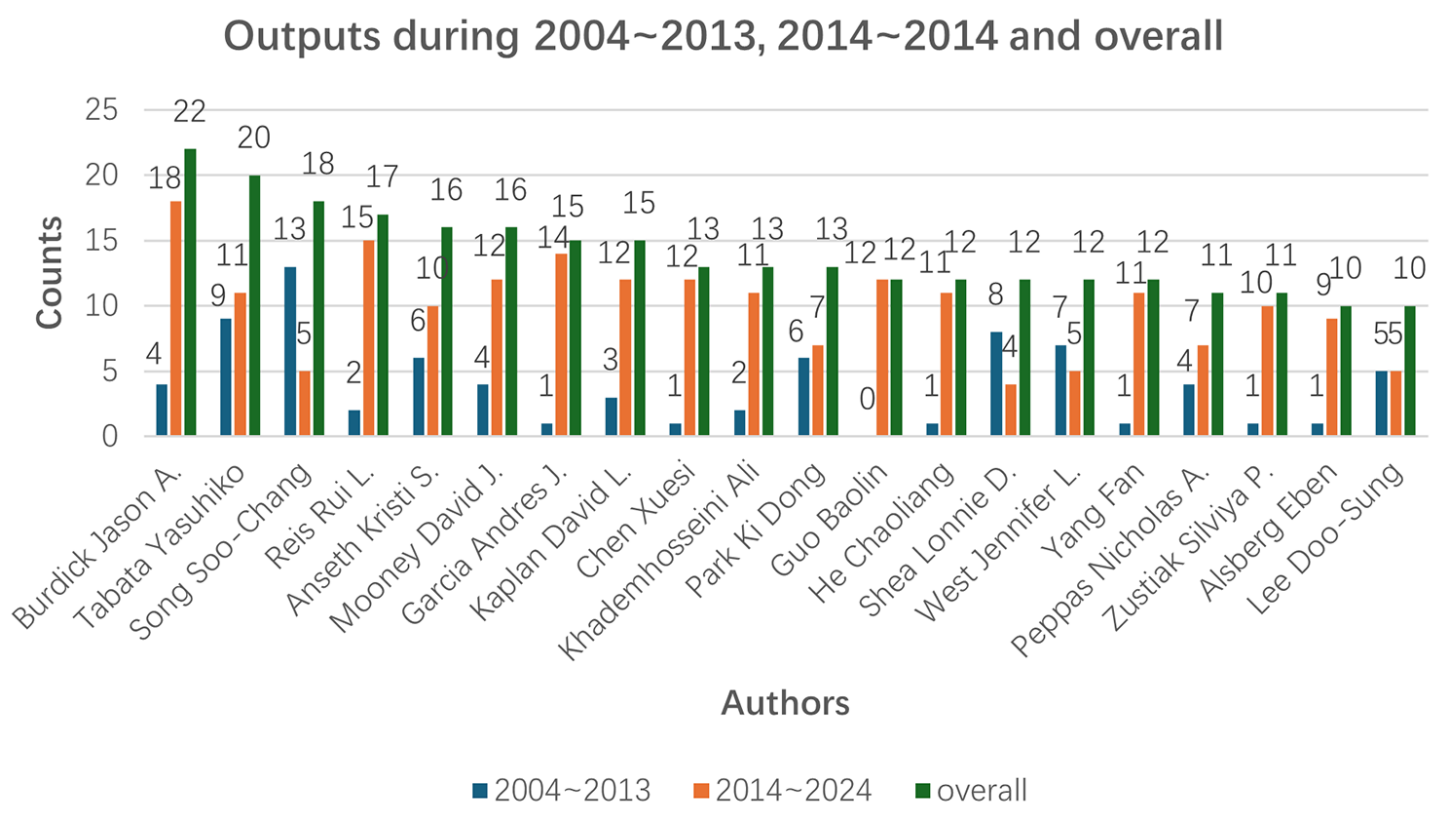
Outputs of the top 20 authors during 2004 ~ 2013, 2014 ~ 2024 and overall

We analyzed the number of articles published by the top 20 authors during 2004–2013 and 2014–2024. Figure 9 shows the outputs of these top 20 authors for 2004–2013, 2014–2024, and overall. The data presented in Fig. 9 demonstrate that most of the top 20 authors have made significant contributions to the relevant literature within the past decade. Notably, three Chinese authors, Chen Xuesi, Guo Baolin, and He Chaoliang, have published papers on the biomedical applications of hydrogels from 2017 to 2023, indicating their active engagement in this research area for approximately five to six years. This observation aligns with previous studies reporting an increase in articles by Chinese researchers after 2016. Additionally, Professor Tabata Yasuhiko from Japan, Professor Song Soo-Chang and Professor Park Ki Dong from South Korea, and Professor Shea Lonnie D. and Professor West Jennifer L. from the United States have made substantial contributions between 2004 and 2013 regarding the biomedical applications of hydrogels, highlighting Japan, South Korea, and the USA as pioneering nations that laid a solid foundation for subsequent investigations.

**Table 5:** Top 20 authors in the field of hydrogel biomedical application (1973–2024)

Number	Authors	Counts	Affiliations	Countries / Regions
1	Burdick Jason A.	22	University of Colorado Boulder	USA
2	Tabata Yasuhiko	20	Kyoto University	Japan
3	Song Soo-Chang	18	Korea Institute of	Korea
			Science and Technology	
4	Reis Rui L.	17	Universidade do Minho	Portugal
5	Anseth Kristi S.	16	University of Colorado	USA
6	Mooney David J.	16	Harvard University	USA
7	Garcia Andres J.	15	George W. Woodruff School	USA
			of Mechanical Engineering	
8	Kaplan David L.	15	Tufts University	USA
9	Chen Xuesi	13	Changchun Institute of	People R China
			Applied Chemistry	
			Chinese Academy of Sciences	
10	Khademhosseini Ali	13	University of Colorado Boulder	USA
11	Park Ki Dong	13	Ajou University	Korea
12	Guo Baolin	12	Xi’an Jiaotong University	People R China
13	He Chaoliang	12	Changchun Institute of	People R China
			Applied Chemistry	
			Chinese Academy of Sciences	
14	Shea Lonnie D.	12	University of Michigan	USA
15	West Jennifer L.	12	Duke University	USA
16	Yang Fan	12	Stanford University	USA
17	Peppas Nicholas A.	11	University of Texas at Austin	USA
18	Zustiak Silviya P.	11	Saint Louis University	USA
19	Alsberg Eben	10	University of Illinois Chicago	USA
20	Lee Doo-Sung	10	Sungkyunkwan University	Korea

**Fig. 10:**
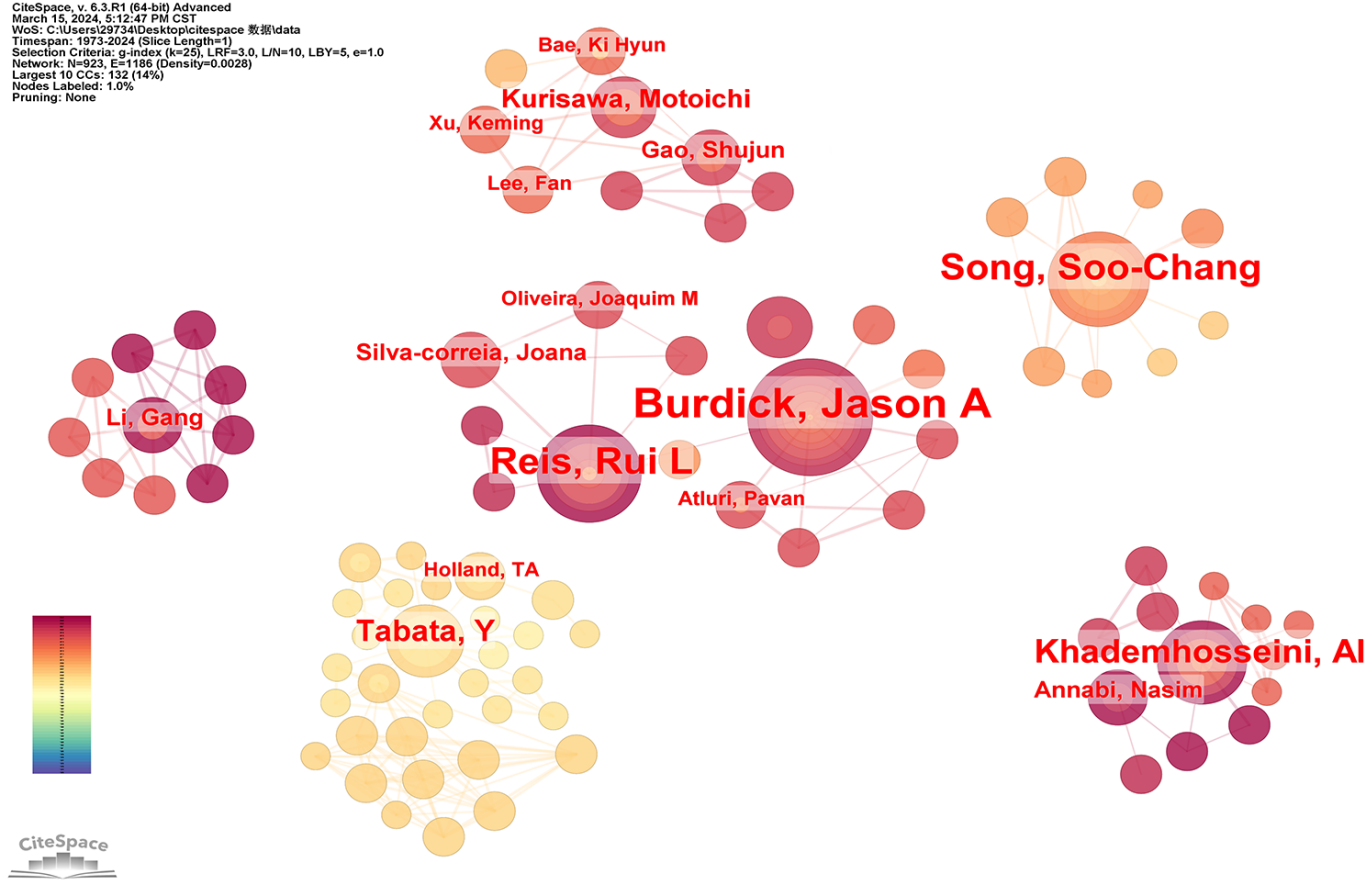
Network diagram between authors in the field of hydrogel biomedical application

We determined the dispersion and distribution of academics’ collaboration networks by examining their cooperative relationship. (Fig. 10). In contrast to the cohesive national and institutional collaborative networks, the authors’ collaborative networks exhibited a dispersed pattern. A limited number of scholars maintained a closely-knit cooperative relationship primarily based on geographical proximity. For example, Burdick Jason A from the University of Pennsylvania and Reis Rui L from the University of Minho both had strong cooperation with other researchers, and their articles were often referenced. It is worth noting that Burdick Jason A mostly cooperated with researchers from the USA, while Reis Rui L largely cooperated with researchers from Portugal. Tabata Yasuhiko from Kyoto University mostly cooperated with researchers from Japan and Song Soo-Chang from Korea Institute of Science and Technology cooperated with researchers from Korea. The cooperation among authors was predominantly influenced by geographical proximity, as researchers tend to collaborate more frequently with colleagues from their own country. In the future, we anticipate an upsurge in innovative collaborations across nations.

### Analysis of discipline evolution

**Fig. 11:**
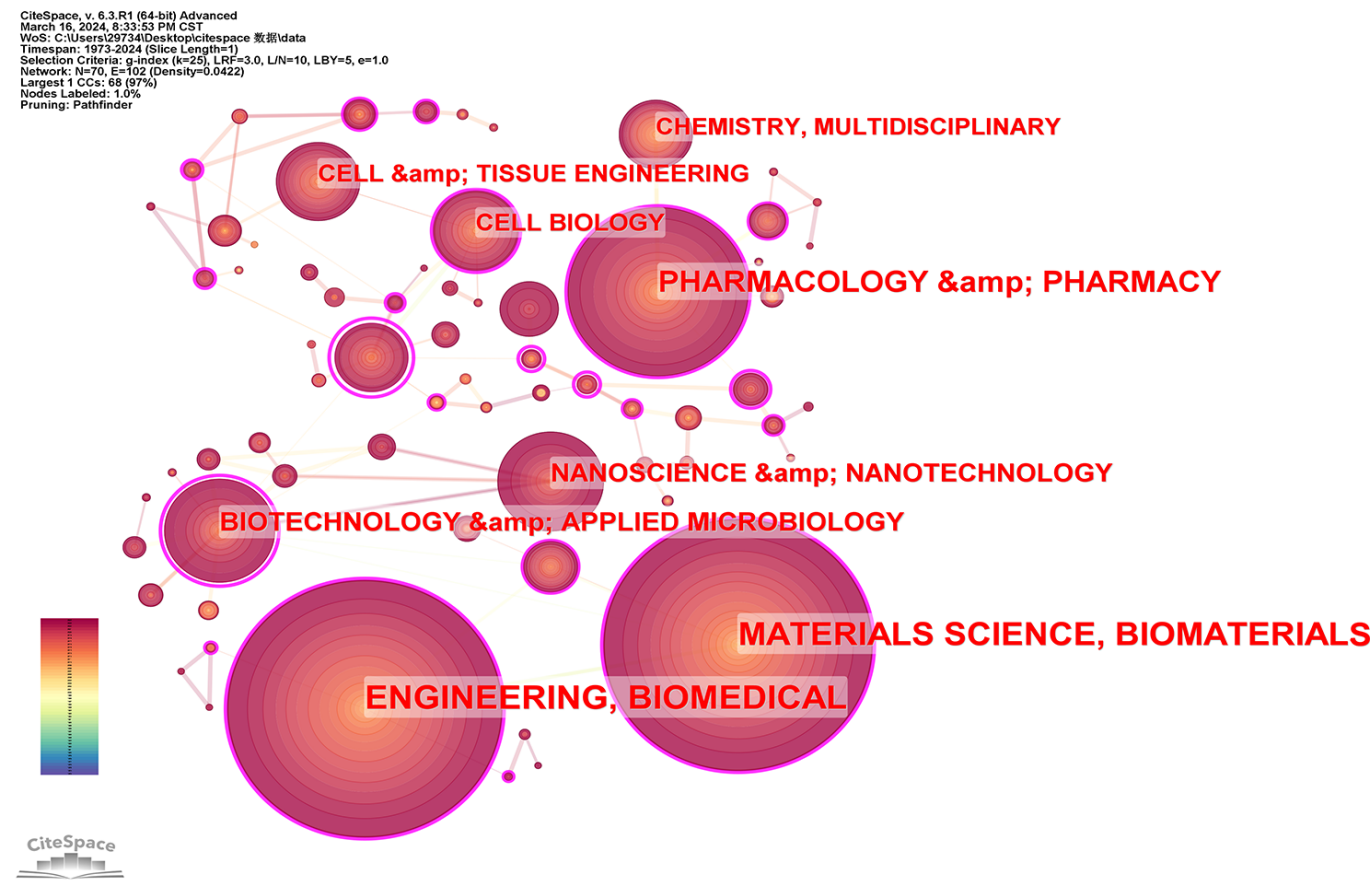
Disciplinary network in the field of hydrogel biomedical application from1973 to 2024

Through comprehensive analysis across various disciplines, we established an intricate network of hydrogel biomedical applications. Figure 11 visually depicts the progression of both mainstream and interdisciplinary fields in this domain, underscoring the extensive coverage of hydrogel biomedical applications across multiple areas. This can be attributed to their significant impact and widespread research focus. Engineering and Biomedical are the main subjects, this is because we are concerned with the biomedical application of hydrogel. The second is Materials Science and Biomaterials, this is because hydrogels belong to a special kind of material, and the advancement of biomaterials has the potential to broaden the scope of hydrogel applications in the field of biomedicine. Pharmacology & Pharmacy and Biotechnology & Applied Microbiology are also important disciplines in this field. From this, it can be inferred that the biomedical application of hydrogels is closely intertwined with pharmacology due to their exceptional performance as drug carriers.

**Fig. 12:**
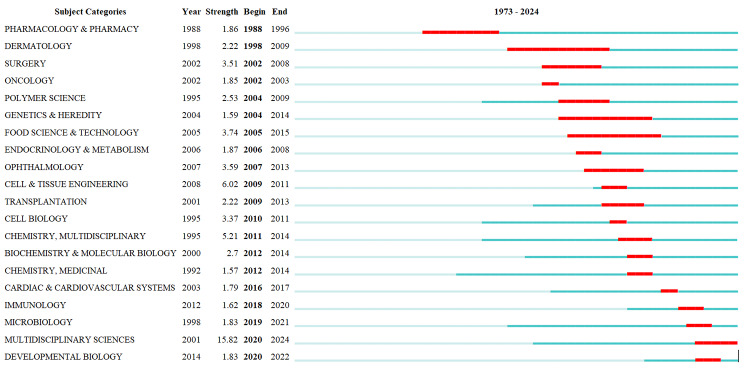
Brust disciplines in the field of hydrogel biomedical application during 1973 ~ 2024

Moreover, hydrogels exhibit diverse applications in various biotechnologies and demonstrate promising potential against microbial infections. The disciplines that occurred from 1973 to 2024 are included in Fig. 12, which amply illustrates the diverse nature of hydrogel biomedical applications. According to the data, there is a discipline burst in the field of hydrogel biomedical application every 1 ~ 2 years on average; nevertheless, the duration of the developing disciplines burst prior to 2005 is very long. However, the duration of disciplines burst after that is much shorter. This indicates that over the past ten years, there was a growing frequency in the emergence of novel disciplines and continuous identification of research frontiers. Consequently, researchers are urged to diligently monitor the research frontiers to avoid overlooking current trends. Among them, burst disciplines DERMATOLOGY, GENETICS & HEREDITY, PHARMACOLOGY & PHARMACY and FOOD SCIENCE & TECHNOLOGY maintain the longest duration, which is about 10 years, the shortest is ONCOLOGY, CELL BIOLOGY, and CARDIAC & CARDIOVASCULAR SYSTEM, which lasted only one year. The focus of these disciplines lies in the specialized domains of hydrogel medical applications, resulting in a limited number of research innovations. Consequently, the majority of valuable research content will be thoroughly investigated within a span of one to two years.

### Analysis of hotspot evolution

#### Keywords co-occurrence analysis

**Fig. 13:**
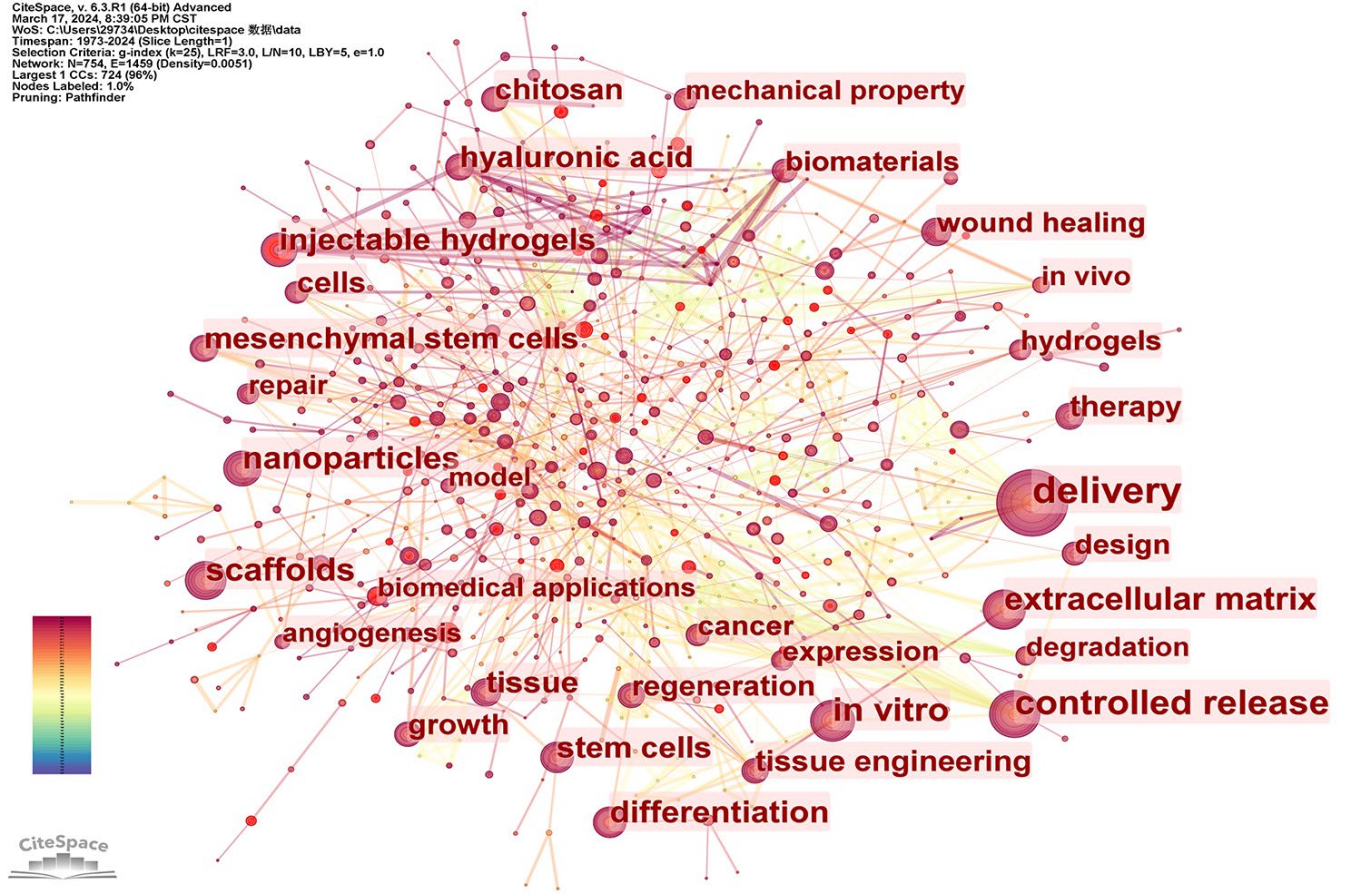
Co-occurrence keyword network in the field of hydrogel biomedical application. (1973 ~ 2024)

Keyword co-occurrence serves as an effective means to reflect the research hotspots within a specific field, while emergent keywords can indicate cutting-edge topics. When analyzing the dispersion of keywords, our initial focus lies on examining the phenomenon of keyword co-occurrence. As depicted in Fig. 13, diverse nodes represent various keywords, with node size denoting the number of associated articles, and lines between nodes representing relationships among these keywords. The intricate interconnections among nodes suggest complex associations. The top ten keywords include delivery, controlled release, in vitro, scaffolds, nanoparticles, differentiation, extracellular matrix, hyaluronic acid, tissue, and mesenchymal stem cells. Among them, delivery is the one with the biggest node and the most intricate web of connecting links.

**Fig. 14:**
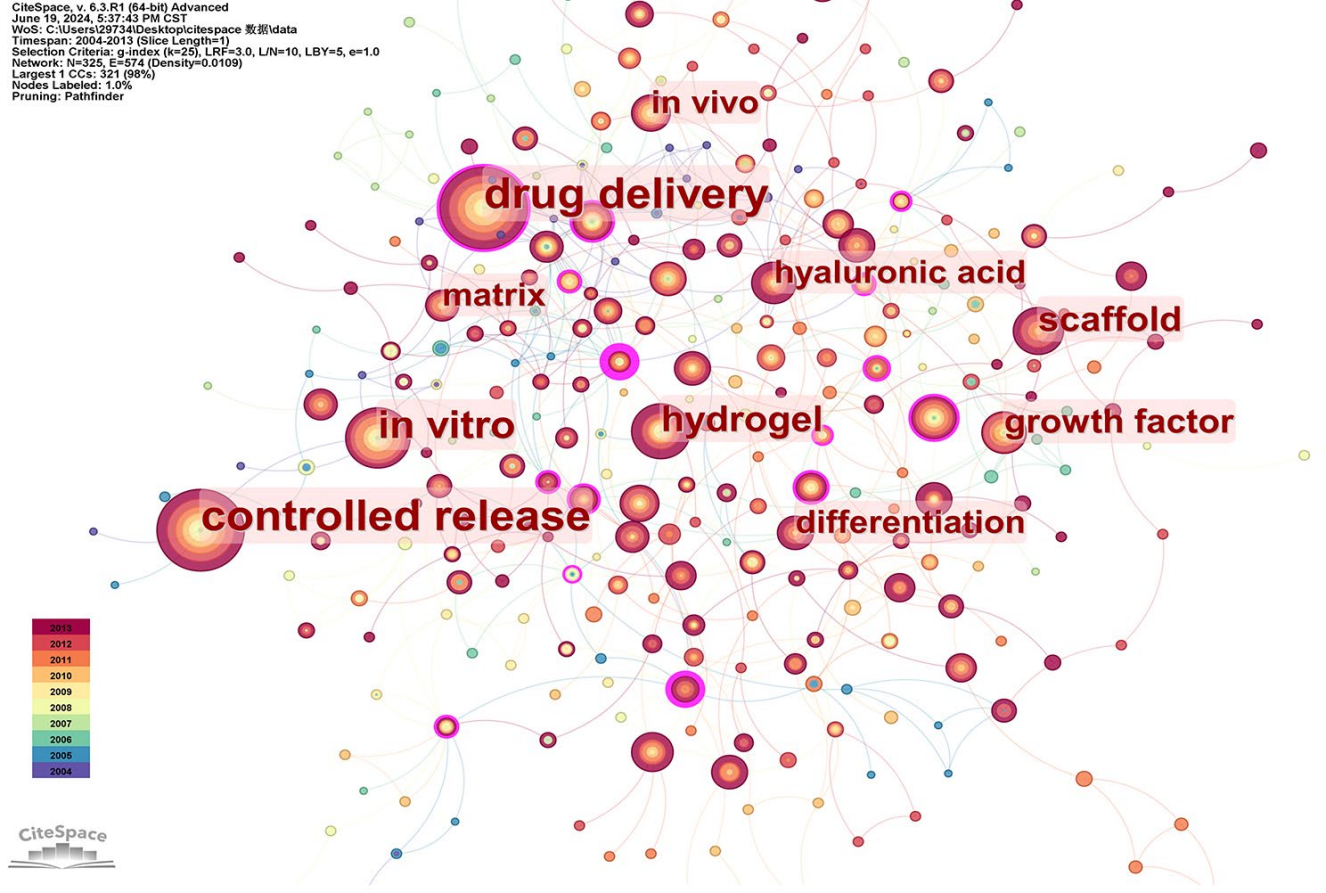
Co-occurrence keyword network in the field of hydrogel biomedical application. (2004 ~ 2013)

**Fig. 15:**
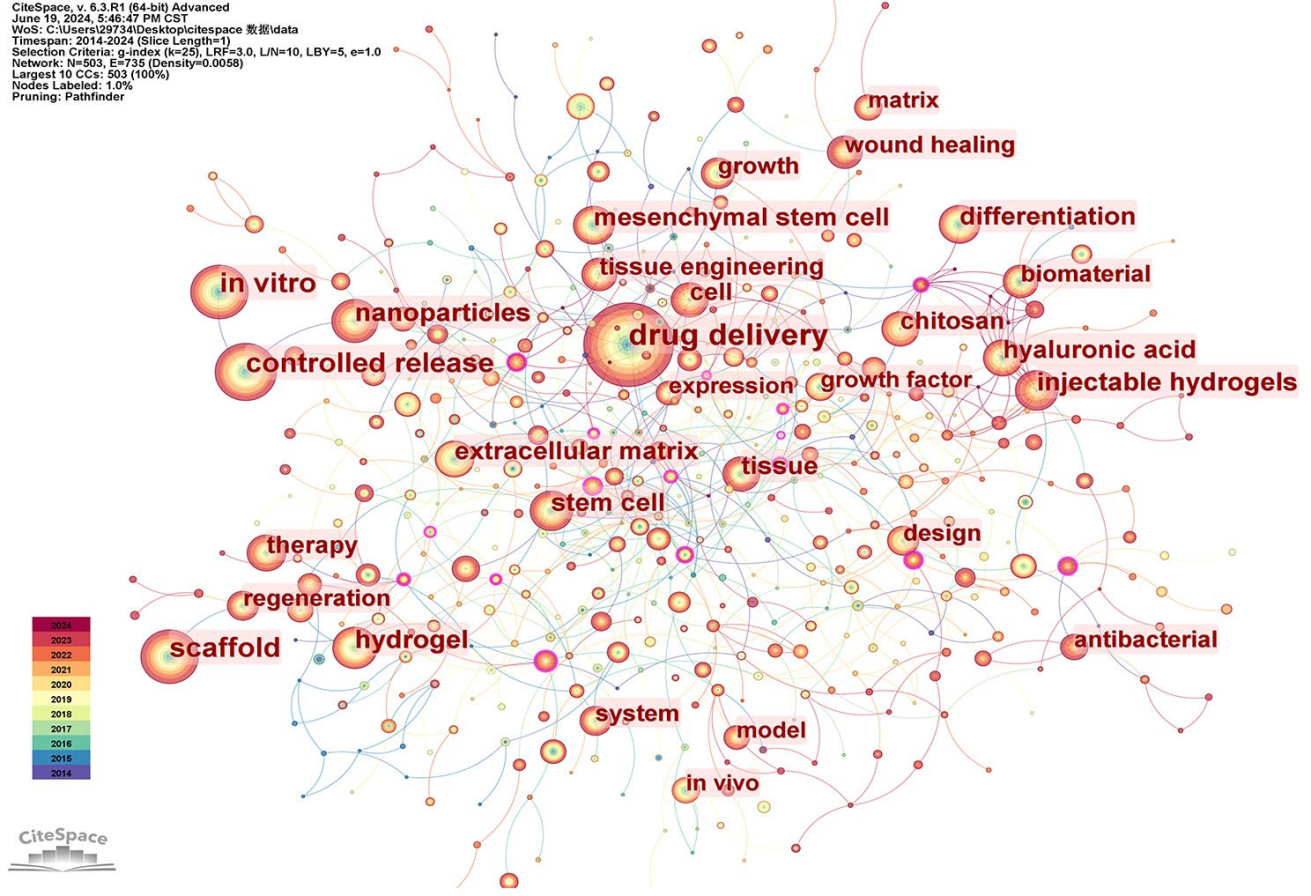
Co-occurrence keyword network in the field of hydrogel biomedical application. (2014 ~ 2024)

Subsequently, we performed keyword co-occurrence analysis on literature spanning the periods 2004 ~ 2013 and 2014 ~ 2024, respectively, yielding the depicted results in the Figs. 14 and 15. The top ten keywords in Fig. 14 include drug delivery, controlled release, in vitro, hydrogel, scaffold, growth factor, matrix, differentiation, in vivo and hyaluronic acid. The top ten keywords in Fig. 15 include drug delivery, controlled release, scaffold, in vitro, nanoparticles, hydrogel, injectable hydrogels, stem cell, mesenchymal stem cell and differentiation. Drug delivery and controlled release are frequently mentioned in the three images, indicating that drug delivery has always been a key application field for hydrogels. Additionally, scaffolds are also prominently featured, demonstrating the widespread use of hydrogels in biological scaffold production. In the past decade, new keywords such as nanoparticles have emerged within the top ten, suggesting the emergence of new research areas. For future biomedical research on hydrogel applications and development of new hydrogels, greater attention should be given to nano-hydrogel development and application as well as injectable hydrogels. Furthermore, in the field of hydrogel applications, more emphasis should be placed on utilizing novel hydrogels with stem cells, particularly mesenchymal stem cells.

#### Keywords cluster analysis

**Fig. 16:**
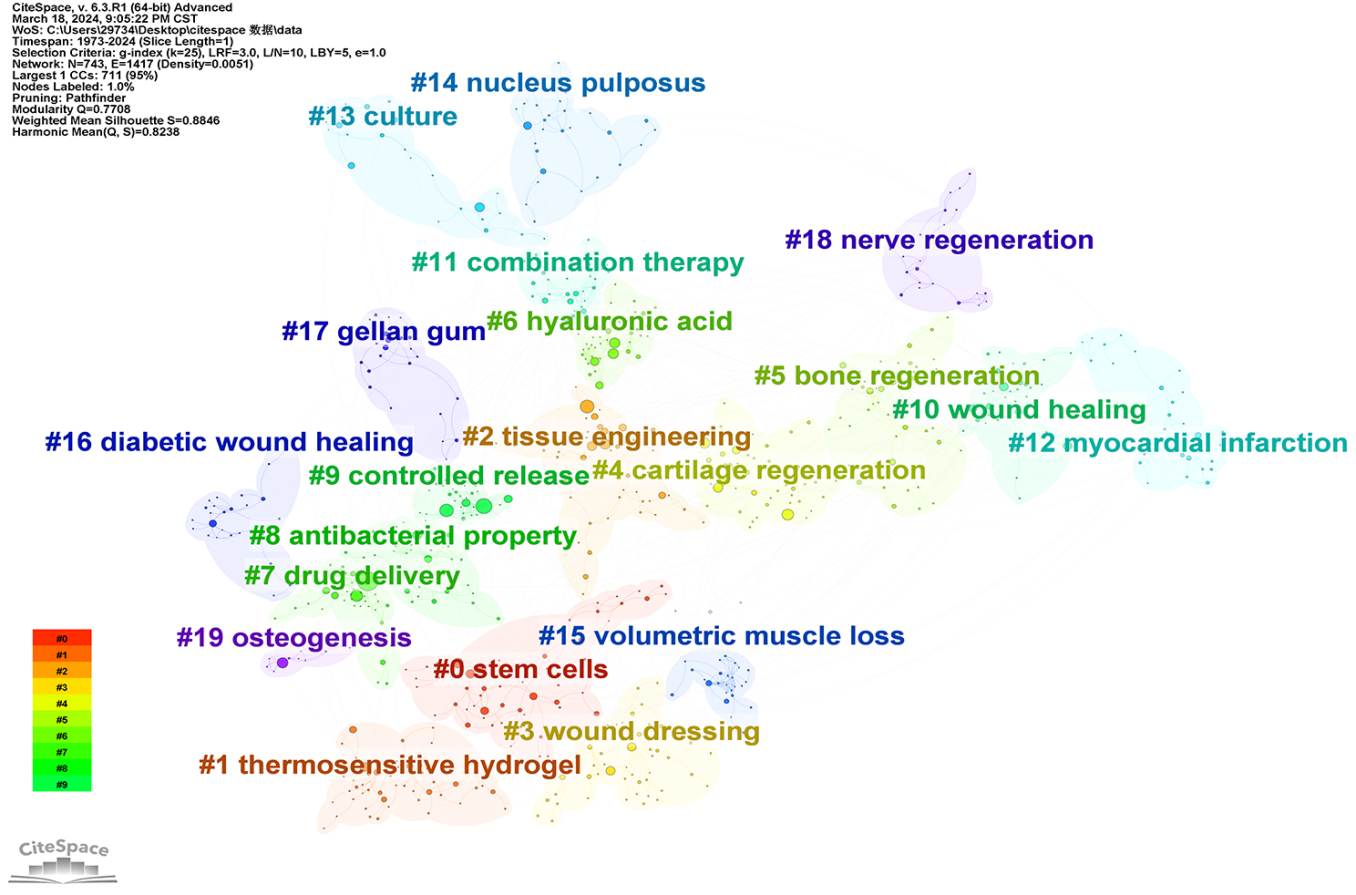
Co-occurrence clustering keyword network in the field of hydrogel biomedical application. (1973 ~ 2024)

Cluster analysis of keywords was performed on all articles (1973 ~ 2024) within the hydrogel biomedical application field (Fig. 16). Keywords exhibiting similarities were grouped, while distinct clusters were formed for each keyword. The number of keywords within each cluster decreased as the sub-cluster number increased, with each cluster comprising multiple closely associated terms. Furthermore, co-occurring keywords were categorized into 20 sub-clusters, denoted by numbers ranging from 0 to 19, including #0 stem cells, #2 tissue engineering, #3 wound dressing, #4 cartilage regeneration, #5 bone generation, #6 hyaluronic acid, #7 drug delivery, #8 antibacterial property, #9 controlled release, #10 wound healing, #11 combination therapy, #12 myocardial infarction, #13 culture, #14 nucleus pulposus, #15 volumetric muscle loss, #16 diabetic wound healing, #17 gellan gum, #18 nerve regeneration, #19 osteogenesis.

**Fig. 17:**
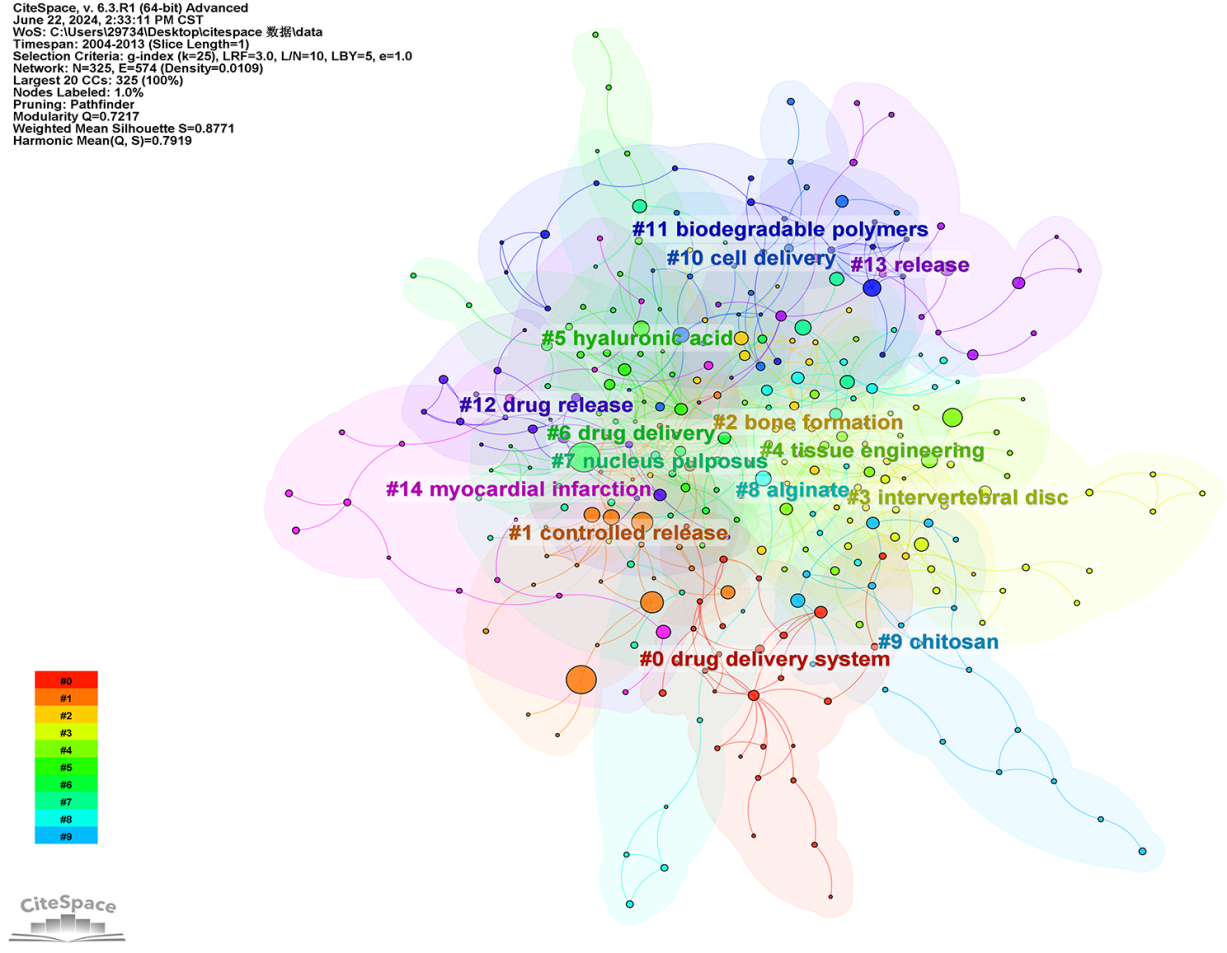
Co-occurrence clustering keyword network in the field of hydrogel biomedical application. (2004 ~ 2013)

**Fig. 18:**
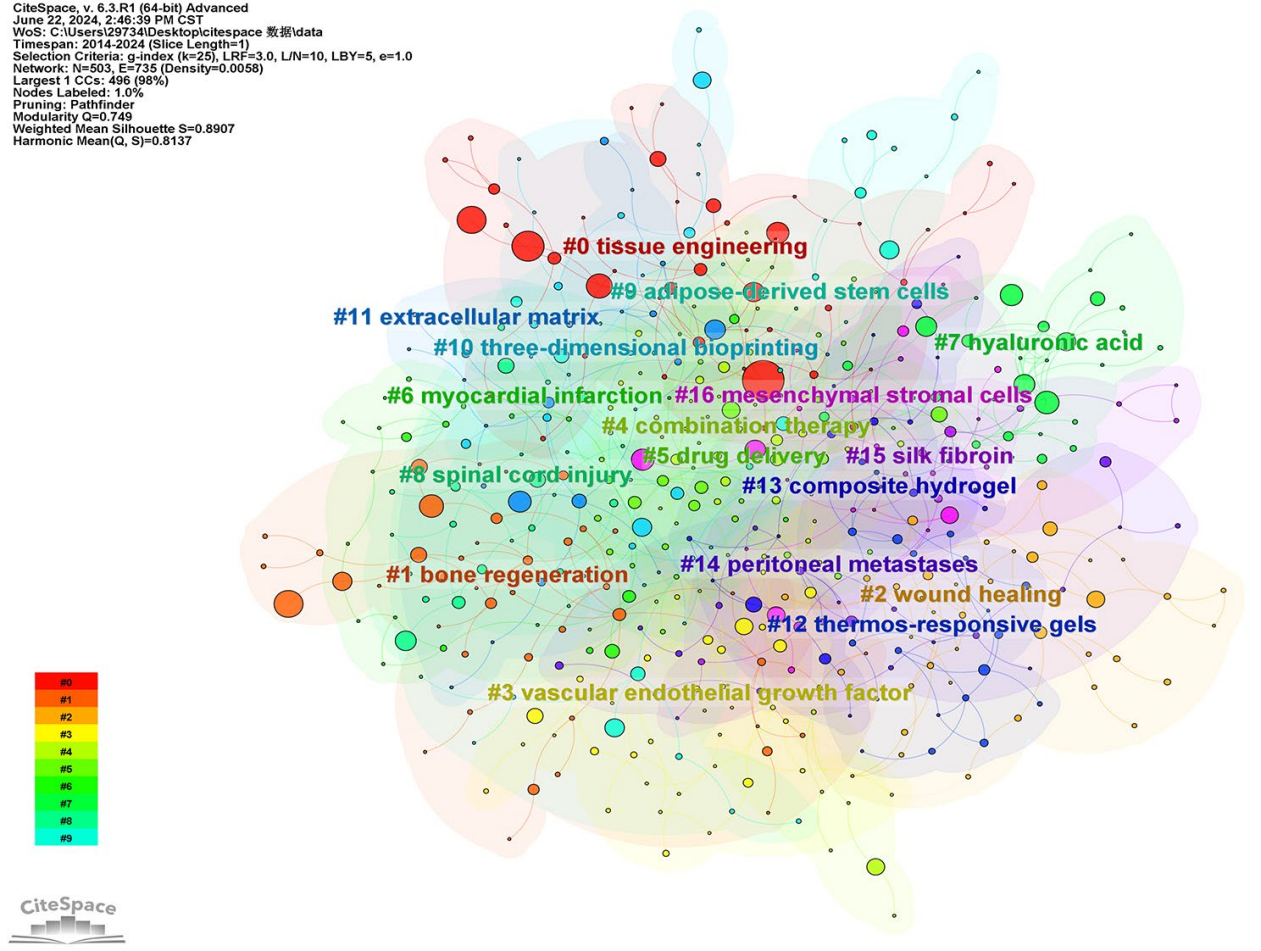
Co-occurrence clustering keyword network in the field of hydrogel biomedical application. (2014 ~ 2024)

We also performed cluster analysis on literature spanning the periods 2004–2013 and 2014–2024. Figures 17 and 18 present the results. The keywords in articles published during 2004–2013 can be grouped into 15 clusters: #1 drug delivery system, #1 controlled release, #2 bone formation, #3 intervertebral disc, #4 tissue engineering, #5 hyaluronic acid, #6 drug delivery, #7 nucleus pulposus, #8 alginate, #9 chitosan, #10 cell delivery, #11 biodegradable polymers, #12 drug release, #13 release, #14 myocardial infarction. For the period 2014–2024, the keywords can be grouped into 17 clusters: #0 tissue engineering, #1 bone regeneration, #2 wound healing, #3 vascular endothelial growth factor, #4 combination therapy, #5 drug delivery, #6 myocardial infarction, #7 hyaluronic acid, #8 spinal cord injury, #9 adipose-derived stem cells, #10 three-dimensional bioprinting, #11 extracellular matrix, #12 thermos-responsive gels, #13 composite hydrogel, #14 peritoneal metastases, #15 skin fibroin, #16 mesenchymal stromal cells. Through tertiary cluster analysis, we identified recurring labels for hydrogel applications, including tissue engineering, controlled release, drug delivery, and hyaluronic acid. These reiterated labels accurately reflect the principal domains where hydrogels are employed. Notably, wound healing and bone regeneration have emerged as significant applications in the past decade. These two areas encompass numerous keywords, indicating a heightened research focus on utilizing hydrogels for these purposes in recent years. This also suggests potential directions for future investigations.

**Fig. 19:**
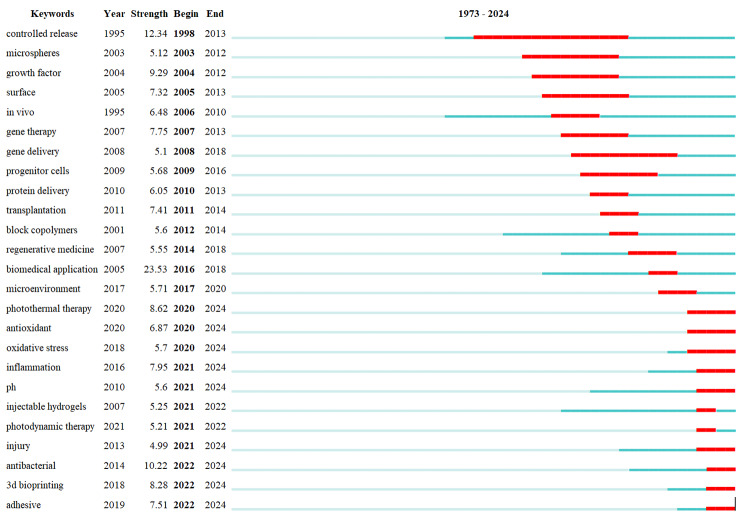
Top 25 bursting keywords

Bursting keywords are frequently referenced over a specific period. CiteSpace can identify bursting keywords, which serve as a foundation for assessing the forefront of research. We used Fig. 19 to show our results. The timeline is depicted by a green line, with the bold red section indicating the temporal phase of the keyword outbreak. It signifies the year when the keyword originated, ceased, and its duration of prominence. The top 25 keywords based on their duration include controlled release (1998 ~ 2013), microspheres (2003 ~ 2012), growth factor (2004 ~ 2012), surface (2005 ~ 2013), in vivo (2006 ~ 2010), gene therapy (2007 ~ 2013), gene delivery (2008 ~ 2018), progenitor cells (2009 ~ 2016), protein delivery (2010 ~ 2013), transplantation (2011 ~ 2014), block copolymers (2012 ~ 2014), regenerative medicine (2014 ~ 2018), biomedical application (2016 ~ 2018), microenvironment (2017 ~ 2020), photothermal therapy (2020 ~ 2024), antioxidant (2020 ~ 2024), oxidative stress (2020 ~ 2024), inflammation (2021 ~ 2024), PH (2021 ~ 2024), injectable hydrogels (2021 ~ 2022), photodynamic therapy (2021 ~ 2022), injury (2021 ~ 2024), antibacterial (2022 ~ 2024), 3d bioprinting (2022 ~ 2024) and adhesive (2022 ~ 2024).

### Analysis of co-citation

#### Analysis of author co-citation

**Fig. 20:**
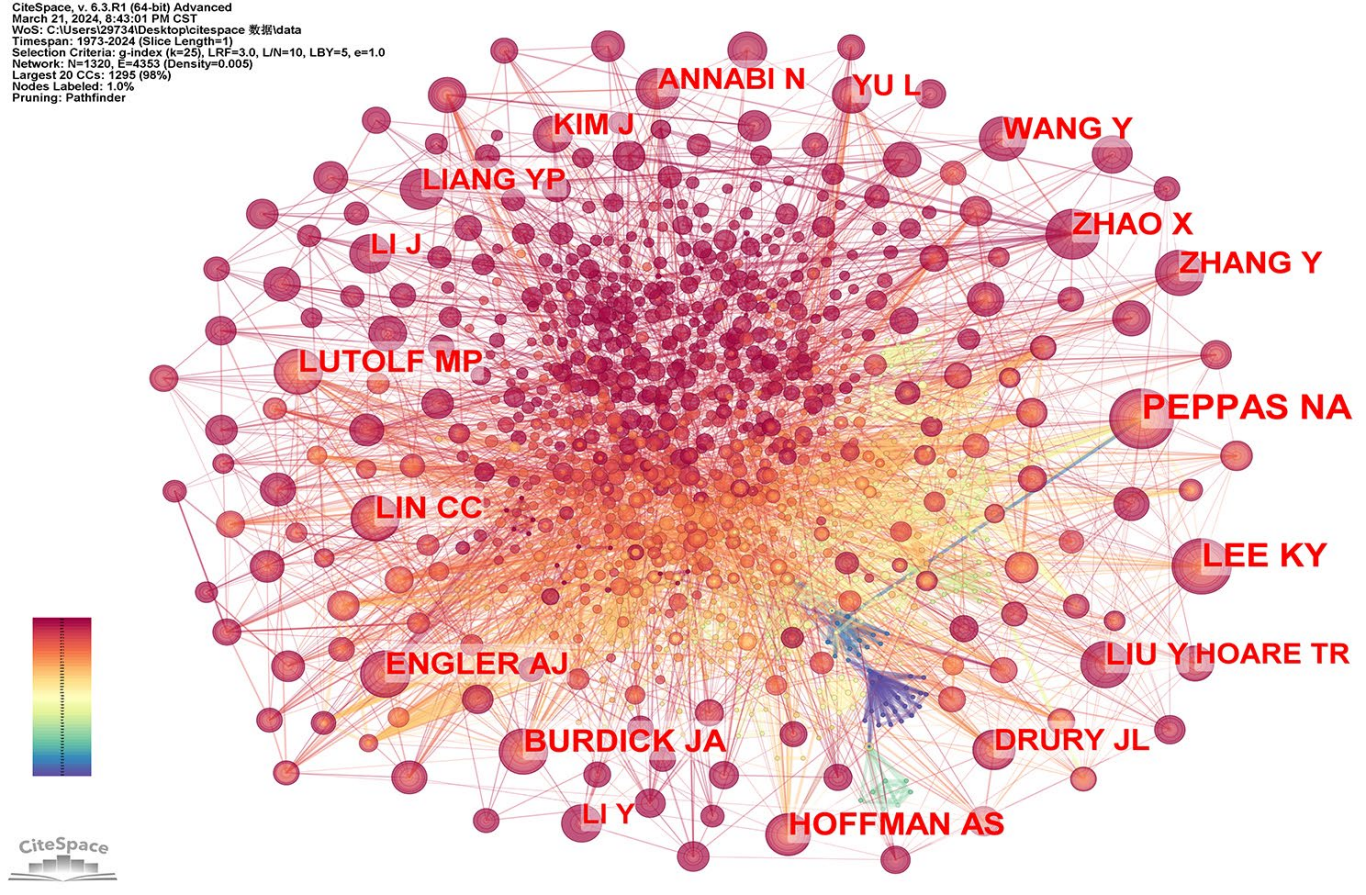
Author co-citation analysis in the hydrogel biomedical application field

We utilized CiteSpace software to analyze a total of 2862 articles published between 1973 and 2024, employing a time slice of one year. From each time slice, we selected the most frequently cited or referenced entries. Figure 20 presents the co-citation network diagram of the author, comprising 1320 nodes and 4353 lines. These nodes represent the references under analysis while the lines depict co-citation relationships among documents. Larger nodes indicate studies that have been cited more frequently over time. The amounts of citations in various periods are indicated by the color and thickness of the circles within the nodes. The colors of the lines match the time slices exactly; the more recent years are represented by warmer hues and the earlier years by cooler hues. The first-ranked authors are PEPPAS NA et al. with a citation number of 278, followed by LEE KY et al. with a citation number of 214, BURDICK JA et al. with a citation number of 155, and HOFFMAN AS with a citation number of 149.

Nicholas A. Peppas is a distinguished chair professor at the University of Texas at Austin. He spearheaded groundbreaking advancements in biomaterials, nanomaterials, polymer physics, drug delivery, and bio-nanotechnology through his multidisciplinary approach. Professor Peppas established fundamental principles and a rational design methodology for biomedical systems while also developing controlled-release devices and models for drug and protein diffusion in biological tissues. His exceptional contributions to the fields of biomaterials, controlled drug release, and bio-nanotechnology have earned him global recognition as one of the foremost scientists in these areas.

#### Analysis of document co-citation

**Fig. 21:**
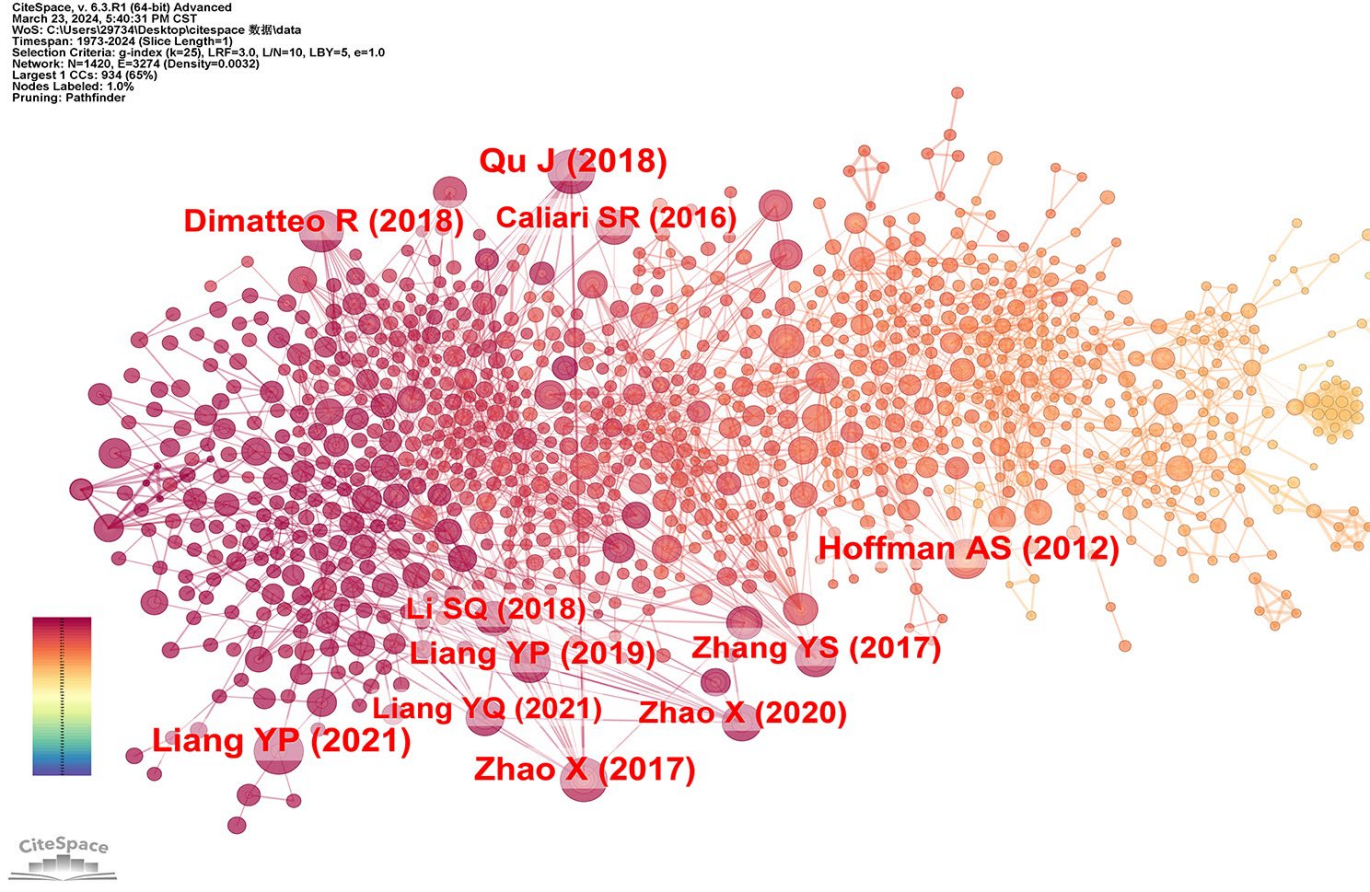
Document co-citation analysis in hydrogel biomedical application

We used CiteSpace software to analyze a total of 2862 articles published between 1973 and 2024, with a time slice of one year. Figure 21 shows the visualization analysis results. Table 6 shows the Top 10 ranked articles by co-citation counts. The top-ranked document by citation counts is Jin Qu’s article with 48 citation counts: “Antibacterial adhesive injectable hydrogels with rapid self-healing, extensibility, and compressibility as wound dressing for joints skin wound healing” (DOI: 10.1016/j.biomaterials.2018.08.044). The first author is Jin Qu from the State Key Laboratory for Mechanical Behavior of Materials, Frontier Institute of Science and Technology, Xi’an Jiao Tong University, China. In this article, the authors introduce an injectable hydrogel possessing antibacterial adhesion properties and appropriate mechanical properties, which can serve as a wound dressing for joint or skin healing. A series of hydrogels were synthesized by cross-linking dynamic Schiff bases and co-polymer micelles within a single system and mixture of quaternized chitosan (QCS) and benzaldehyde-terminated PluronicF127 (PF127-CHO) under physiological conditions in this study. These hydrogels exhibited desirable tensile and compressive properties, with a semblable modulus compared to that of human skin, excellent adhesion, and rapid self-healing ability. Moreover, they also demonstrated favorable hemostatic characteristics and excellent biocompatibility. Additionally, curcumin was incorporated into the hydrogel matrix, displaying remarkable antioxidant capacity along with pH-responsive release behavior. In vivo experiments conducted on a full-thickness skin defect model revealed that the curcumin-loaded hydrogel significantly accelerated the wound healing process while promoting increased thickness of granulation tissue deposition and collagen synthesis; furthermore, it upregulated vascular endothelial growth factor (VEGF) levels in the treated area. Consequently, this antibacterial adhesive hydrogel dressing possessing self-healing capability alongside superior mechanical properties exhibits immense potential for application in joint skin wound healing [21].

**Table 6:** Top 10 co-cited documents in hydrogel biomedical application

Numbers	Counts	Author	Affiliation	Countries / Regions	DOI
1	48	Qu J	Xi’an Jiaotong University	PEOPLES R CHINA	10.1016/j.biomaterials.2018.08.044
2	46	Liang YP	Xi’an Jiaotong University	PEOPLES R CHINA	10.1021/acsnano.1c04206
3	45	Zhao X	Xi’an Jiaotong University	Peoples R China	10.1016/j.biomaterials.2017.01.011
4	43	Hoffman AS	University of Washington	USA	10.1016/j.addr.2012.09.010
5	38	Liang YP	Xi’an Jiaotong University	PEOPLES R CHINA	10.1002/smll.201900046
6	36	Dimatteo R	University of California Los Angeles	USA	10.1016/j.addr.2018.03.007
7	33	Zhang YS	Harvard Medical School	USA	10.1126/science.aaf3627
8	32	Zhao X	Xi’an Jiaotong University	Peoples R China	10.1002/adfm.201910748
9	29	Li SQ	The First Hospital of Jilin University	PEOPLES R CHINA	10.1002/advs.201700527
10	28	Liang YQ	Xi’an Jiaotong University	PEOPLES R CHINA	10.1021/acsnano.1c00204

### Analysis of patents

The patent data comes from the incoPat technology innovation information platform of Beijing IncoPat Co., Ltd., which is the first patent database in China with independent intellectual property rights. The search mode used was: “TIAB=(hydrogel?) AND (USETT-CLASS1-CN=(“Medicine and medical treatment”)) AND ((USETT-CLASS1-CN=(“disease”))”. We searched 3,469 patents from 1982 to 2024 and visualized the results.

#### Application trend analysis

**Fig. 22:**
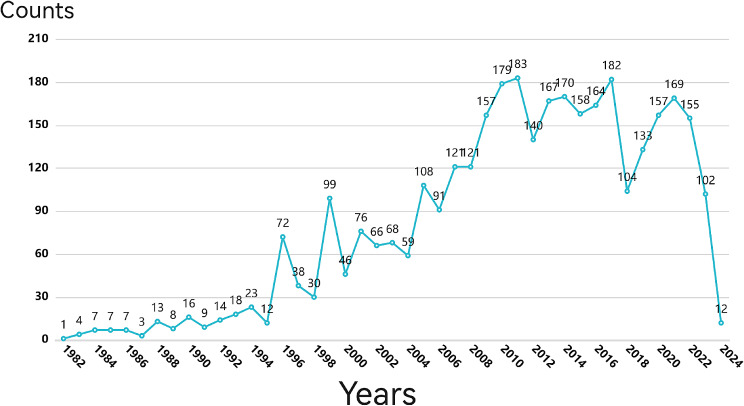
Annual patent applications (1982~2024)

Figure 22 depicts the trend in the number of patent applications, providing a macro-level understanding of their popularity fluctuations over different time periods. The term “number of applications” refers specifically to patents that have been published. It is important to note that general invention patents are disclosed between 3 and 18 months after application, while utility model and design patents are typically disclosed approximately 6 months after application. Overall, the number of patent applications related to hydrogel biomedical applications has shown a growth trend, which can be categorized into three stages: slow development (1982–1995), rapid development (1996–2011), and sustained growth (2012–2024)

The initiation of patent applications related to hydrogel biomedical applications dates back to 1982. From 1982 to 1995, the annual global count for such patent applications remained relatively low, with fewer than 30 filings per year, reflecting a slow pace in technological advancements and a lack of significant industrial scale formation. Since 1996, there has been a substantial increase in the annual global filing rate for patents related to hydrogel biomedical applications. As technology progressed, awareness regarding intellectual property protection among technical experts gradually developed, leading to increased demand. The peak in annual global patent application filings related to hydrogel biomedical applications was observed in 2011, with 183 submissions. From 2012 onwards, the yearly count for global patent applications consistently exceeded 100 as technology matured and stable growth persisted. Particularly during the period from 2012 to 2017, numerous patents emerged within this field, driving technological advancements. Staying updated on these trends will facilitate targeted technological innovation.

#### Inventor analysis

**Fig. 23:**
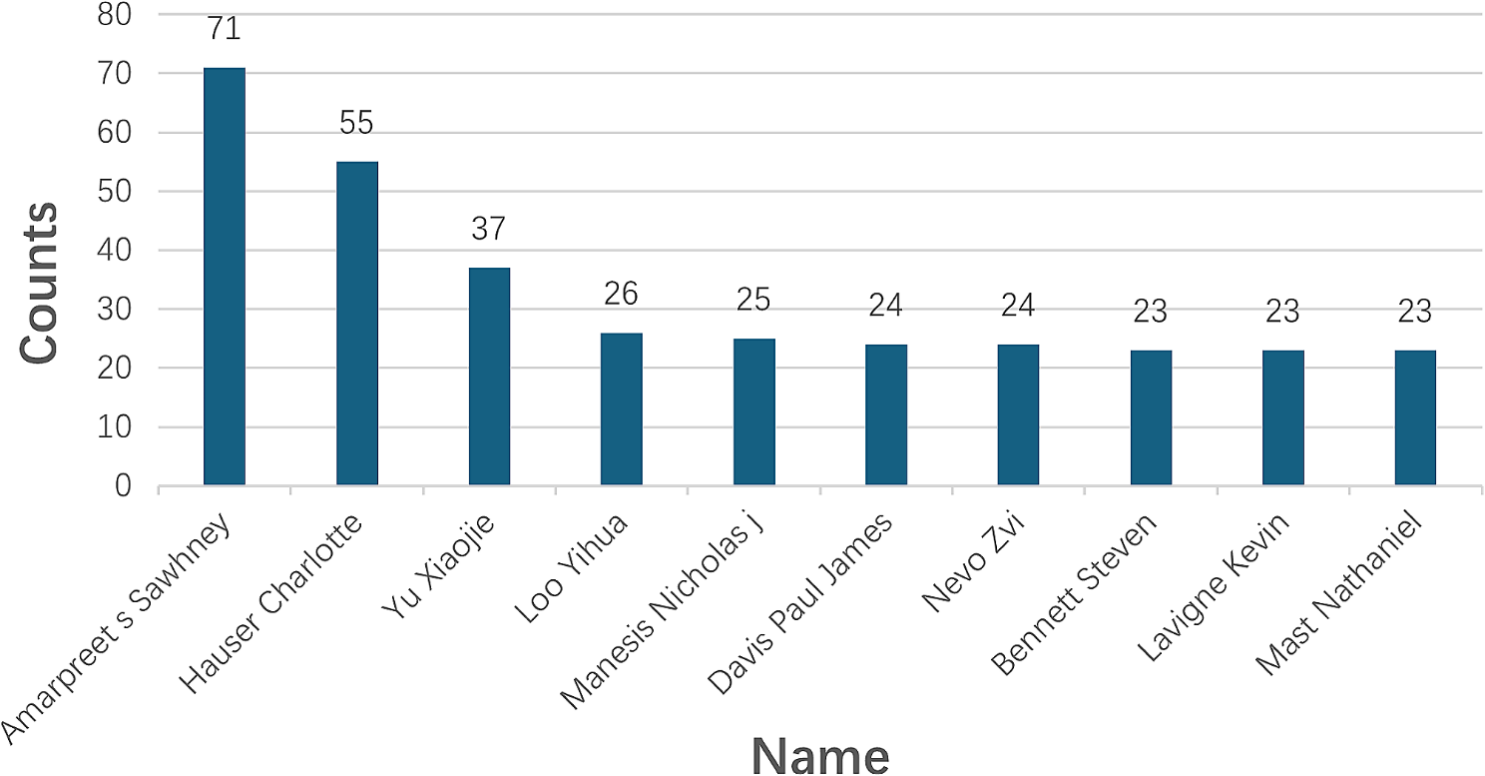
The top 10 inventors with the number of relevant patents

We counted the top 10 inventors based on the number of patents, and the results are shown in Fig. 23. The leading inventor is Amarpreet S. Sawhney from Lexington, Massachusetts, USA, with 71 patents in this field. He is the President and CEO of Ocular Therapeutix, Inc., a company focused on unmet needs in ophthalmic surgical wound management and drug delivery. Following Sawhney are Hauser Charlotte from Singapore with 55 patents, Yu Xiaojie from China with 37 patents, Loo Yihua from Singapore with 26 patents, Manesis Nicholas J. from the USA with 25 patents, Davis Paul James from England with 24 patents, Nevo Zvi from Israel with 24 patents, Bennett Steven from the USA with 23 patents, Lavigne Kevin from the USA with 23 patents, and Mast Nathaniel from the USA with 23 patents. It is noteworthy that half of the top ten inventors are from the United States, indicating that the USA is a major hub for inventions in this field.

#### Global country analysis

**Fig. 24:**
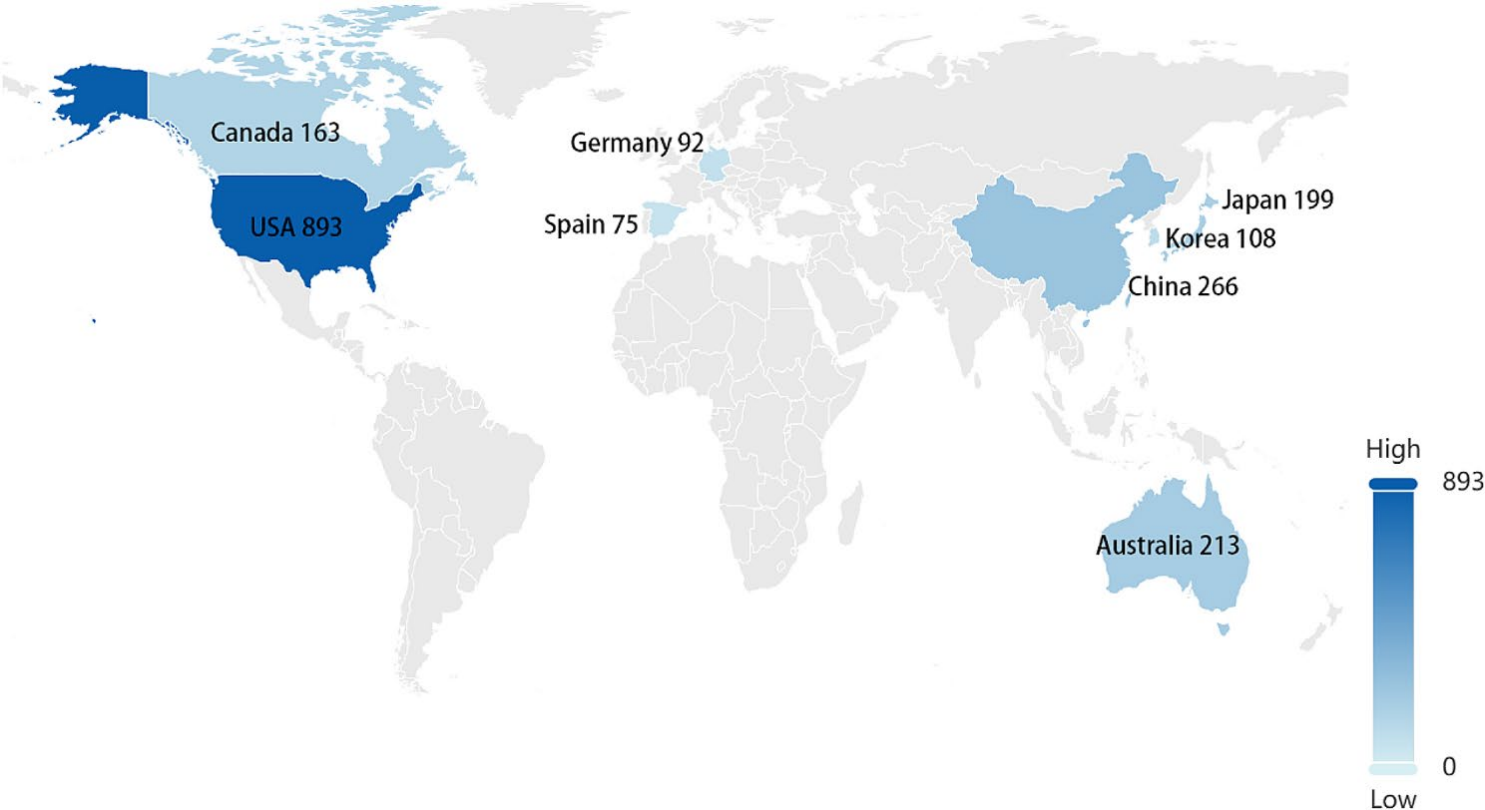
The world’s top eight countries with the number of related patents

We identified the top eight countries in the world based on the number of patents related to the biomedical application of hydrogels: the United States (893), China (266), Australia (213), Japan (199), Canada (163), South Korea (108), Germany (92), and Spain (75). The results are shown in Fig. 24. The United States holds the largest number of patents, reflecting its strong emphasis on safeguarding innovative inventions through a robust patent system. China follows closely, with its relevant authorities continuously refining and enhancing patent-related laws and regulations, addressing loopholes, and fostering a research environment that encourages innovation.

#### Technical application analysis

**Fig. 25:**
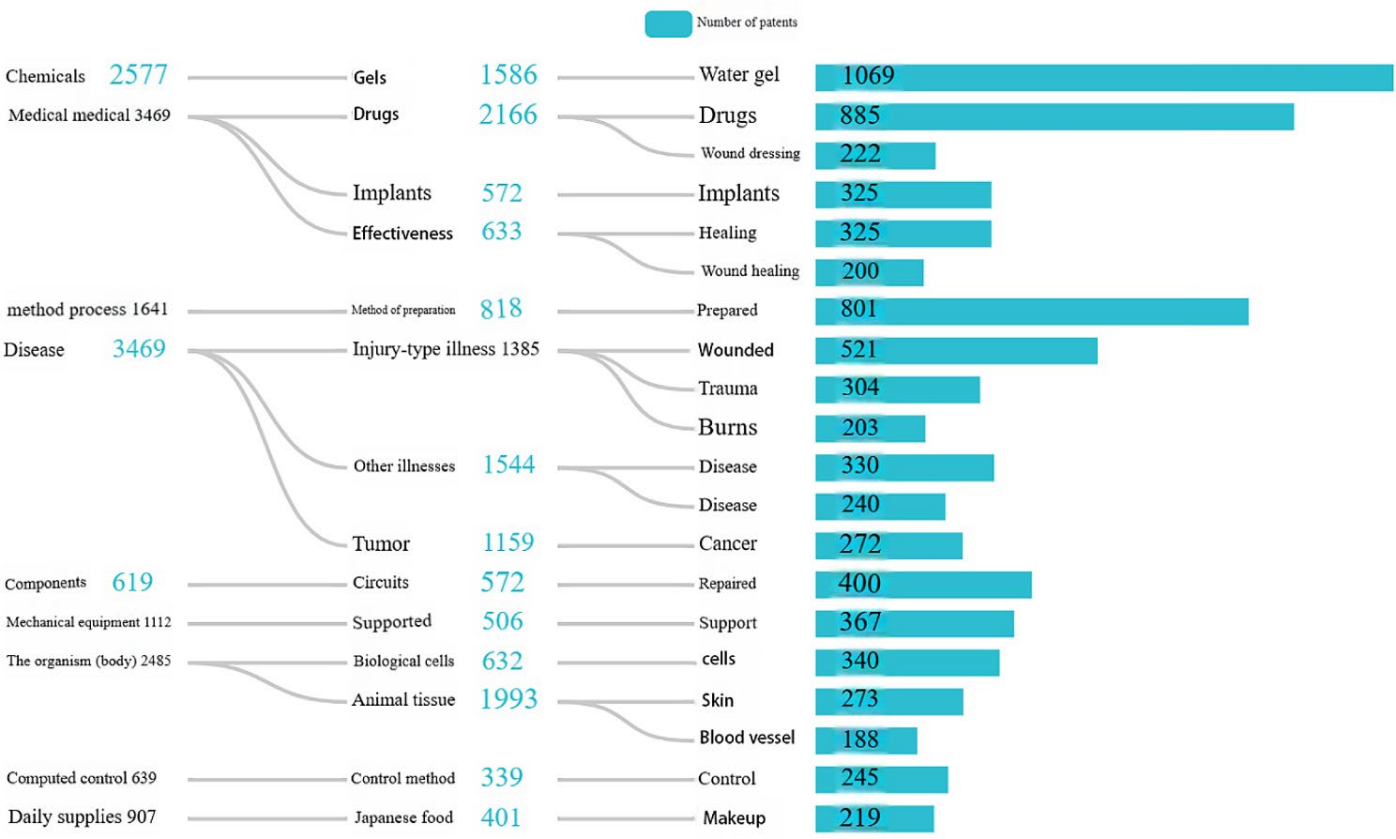
Visual analysis of patents’ technical uses

The analysis function of the incoPat database was utilized to visually depict the primary research domains of pertinent patents in this field. The results are shown in Fig. 25. A meticulous analysis reveals that hydrogel-related patents predominantly focus on drug delivery applications. Most patents combine a specific hydrogel with pharmaceutical agents to achieve optimal delivery efficacy. Additionally, many patents are dedicated to wound healing, where inventors have developed various hydrogel dressings that expedite wound repair and facilitate recovery processes. Furthermore, several related patents have been conceived within the realms of controlled release systems and biological scaffolds. These findings support prior observations regarding the extensive utilization of hydrogels in biomedical applications.

## Discussion

### Research hotspots detection by analysis of highly cited articles

We conducted a comprehensive analysis and interpretation of the highly cited articles, categorizing the utilization of hydrogels in biomedicine into four primary domains: tissue engineering, regeneration medicine, drug delivery & controlled release, and antibacterial material.

#### Hydrogels can be used for tissue engineering

Jeanie L Drury’s article “Hydrogels for Tissue Engineering: Scaffold Design Variables and applications” was highly cited 110 times in this field. This article was published by Biomaterials in 2003. Apart from this, K Y Lee’s article “Hydrogels for tissue engineering” was also cited 94 times in this field. The high citation count of these articles shows that hydrogel was widely used in the tissue engineering field. Jeanie L Drury is a professor working at the University of Michigan. Using concepts from materials science and cell biology, tissue engineering is a young multidisciplinary area that creates artificial tissues or organs in vivo or in vitro [22].

Hydrogels have been explored as promising biomaterials for preventing postoperative adhesion. J.L. Hill-West et al. demonstrated the potential of polyethylene glycol hydrogels in mitigating surgical adhesions [23], while K. Ono et al. proposed chitosan hydrogel as a bioadhesive [24]. Hydrogel scaffolds are also commonly employed for the stabilization and delivery of bioactive molecules, as well as the encapsulation of secretory cells. Presently, systemic administration is predominantly utilized for most small and macromolecular drugs, which not only incurs substantial costs but also pose potential serious side effects. Moreover, numerous bioactive molecules that are essential or advantageous to one tissue may exhibit toxicity towards other tissues. Consequently, hydrogel carriers or scaffolds enabling local and targeted delivery are highly desirable in various scenarios. For example, vascular endothelial growth factor (VEGF), a growth factor that promotes angiogenesis, can be incorporated into ion-cross-linked alginate saline gels, and VEGF is released from the gel by diffusion, mechanical stimulation, or hydrogel degradation [25]. Hydrogel scaffolds also exhibit immense potential for application in cell delivery and tissue development. This is because it can serve as a substrate for facilitating cell adhesion, proliferation, and differentiation.

#### Hydrogels can be used in regeneration medicine

Brandon V. Slaughter’s article “Hydrogels in Regenerative Medicine” was highly cited 81 times in this field. Nowadays, hydrogel scaffolds are extensively employed in the engineering of diverse tissues such as cartilage, bone, muscle, adipose tissue, liver, and neurons. For instance, chondrocytes encapsulated within alginate gel play a pivotal role in the field of cartilage engineering [26]. In addition, hydrogels can also be utilized in 3D cell culture as extracellular matrix substitutes. Hydrogels for cell culture of natural origin typically consist of proteins and extracellular matrix (ECM) components, such as collagen, laminin, fibrin, hyaluronic acid, chitosan, etc. Due to their inherent biocompatibility and bioactivity resulting from the presence of endogenous factors, these hydrogels promote cell survival, proliferation, functional realization, and development. For instance, type I collagen derived from various biomaterials including bovine skin, rat tail tendon, and human placenta is commonly used as a natural hydrogel for 3D cell culture. It facilitates the three-dimensional growth and differentiation of cells while also regulating gene expression through interactions with integrin receptors [27].

#### Hydrogels can be used for drug delivery and controlled release

Todd R. Hoare’s article “Hydrogels in drug delivery: Progress and challenges” was highly cited 97 times in this field, which shows that hydrogel can be used in the drug delivery field. Interest in using hydrogels for medical administration has grown as a result of their distinctive physical characteristics. Effective drug loading inside the gel matrix is made possible by the easy regulation of the density of crosslinks in the hydrogel matrix, which in turn controls the porosity nature of hydrogels. The controlled release kinetics exhibited by hydrogels ensure a sustained and localized drug concentration, thereby enhancing its therapeutic efficacy. Hydrogels are inherently biocompatible due to their full water content and physicochemical resemblance to the natural extracellular matrix, rendering them suitable for biomedical applications. Furthermore, their deformability allows for facile adherence to non-horizontal surfaces within the human body, facilitating optimal performance [28].

#### Hydrogels can be used as antibacterial material

Xin Zhao’s article “Antibacterial anti-oxidant electroactive injectable hydrogel as self-healing wound dressing with hemostasis and adhesiveness for cutaneous wound healing” was highly cited 60 times in this field, which shows that hydrogel can be used in the antibacterial field. Antibacterial hydrogel is a viable material for localized applications due to its excellent biocompatibility, good mechanical characteristics, and inherent or exogenous antibacterial activity. Their physicochemical characteristics are similar to that of the extracellular matrix, and its porous structure and active groups help with gas exchange, medication loading, nutrition transport, and tissue fluid absorption. The problems of antibiotic resistance, toxicity, and antimicrobial inefficiency that come with using traditional antibiotics are successfully resolved by these hydrogels. Being a unique antibacterial material, they not only greatly extend the antibacterial action but also solve problems with limited selectivity, toxicity, and drug resistance that plague conventional materials. Moreover, the multifunctionality of these hydrogels includes anti-inflammatory, hemostatic, and cell growth-promoting capabilities to meet diverse practical application requirements. This is attributed to the unique advantages offered by antibacterial hydrogels: (1) Topical administration avoids systemic side effects caused by excessive drug dosing. (2) Serving as an innovative drug delivery system, it ensures sustained release of antibacterials, resulting in long-lasting efficacy while preventing the emergence of drug-resistant bacteria. (3) Multiple antibacterial components can be loaded onto a single hydrogel, enabling multiple mechanisms for exerting antimicrobial effects. Bacteria find it challenging to develop simultaneous resistance against multiple antibiotics. (4) The synergistic effect between different components confers broad-spectrum antimicrobial capabilities [29].

### Research hotspots detection by co-citation analysis

Table 6 shows the Top 10 ranked articles selected by co-citation counts. The top-ranked document is Jin Qu’s article. It is worth noting that the corresponding author of this article is Guo Baolin, who is the No.12 author in the field of hydrogel biomedical application, according to Table 5. Among the collection of articles in this field, Guo Baolin has 12 articles about hydrogel biomedical applications.

Apart from the first ranked article, the 2, 3, 5, 8, and 10 ranked articles were all done under his guidance. In other words, Guo Baolin is the corresponding author of these 6 articles. By reading the Top 10 literatures we found that 2 and 5 ranked articles’ first author is Liang Yongping, 3 and 8 ranked articles’ first author is Zhao Xin, 10 ranked article’s first author is Liang Yuqing, they are both Guo Baolin’s students. Given the consistent affirmation of equal contributions by all six articles, it is evident that Guo Baolin holds a position of great influence as a researcher in the hydrogel biomedical application field. Notably, Chinese researchers in this domain are predominantly concentrated within the research group led by Guo Baolin at Xi’an Jiaotong University in China, whose publications were substantially cited and played a pivotal role in this field. Remarkably, among the top ten co-cited articles, 60% originate from China, underscoring the significant discursive power attained by Chinese scholars in this field and their widespread recognition among numerous global academics.

Guo Baolin and his students had been engaged in the development of hydrogel dressings that facilitate wound healing. Qu Jin and his team designed a multifunctional injectable micellar hydrogel composite loaded with curcumin in 2018, which can serve as an effective wound dressing for the repair of joint skin injuries. Experimental results demonstrate that this innovative hydrogel possesses remarkable self-healing properties and exhibits excellent mechanical characteristics, rendering it highly suitable for application as a dressing in the treatment of joint skin wounds [21]. In 2019, Liang Yongping and his partners developed a series of hemostatic, antioxidizing, and antibacterial hydrogels based on hyaluronic acid-graft-dopamine and reduced graphene oxide (rGO) through the H2O2/horseradish peroxidase (HRP) system. Within the hydrogel system, dopamine contributes to antioxidant activity, tissue adhesion, conductivity, hemostasis ability prevention, and promotion of self-healing as well as antimicrobial efficacy. Moreover, the hydrogel also demonstrates sustained drug release capability. By significantly upregulating CD31 growth factor expression in the gel matrix, angiogenesis is enhanced while promoting wound healing through improved thickness and collagen deposition in granulation tissue [30]. No longer after that, in 2021, Liang Yongping’s team prepared hydrogels containing nitric oxide (NO) or oxygen, which can enhance wound healing by promoting blood flow and oxygen supply through the controlled release of NO or oxygen. Moreover, they also explored the incorporation of other functional substances such as mi-RNAs, peptides, and proteins into hydrogels to regulate tissue macrophage polarization, reduce inflammatory responses, promote angiogenesis, and facilitate collagen deposition for improved wound healing outcomes. Additionally, they proposed the concept and application of self-healing hydrogels capable of autonomously repairing both functional and structural damage during the wound-healing process while preventing external bacterial infection [31]. There is no exception that Guo Baolin’s other student Zhao Xin also had made great achievements in the wound healing application of hydrogels. In 2017, an injectable hydrogel with multifunctional properties, such as antibacterial, antioxidant, hemostatic, and adhesive qualities, was created by Xin Zhao and his colleagues as a wound dressing. The researchers utilized a quaternized chitosan-g-polyaniline (QCSP) and crosslinker concentration of 1.5 wt% benzaldehyde group functionalized poly (ethylene glycol)-co-poly (glycerol sebacate) (PEGS-FA) to prepare this hydrogel, which exhibits excellent water solubility, antibacterial activity, electrical conductivity, and free radical eliminating ability. The experimental results demonstrated the hydrogel’s rapid promotion of the healing process, effective hemostasis ability, and strong adhesion. In the animal experiment conducted using a full-thickness skin defect model, the application of the hydrogel dressing significantly enhanced the wound-healing process [29]. In 2020, his team developed a physically dual-network hydrogel exhibiting rapid shape adaptation, quick self-healing, antioxidant properties, and near-infrared/acid-base responsiveness that can be used for the treatment of multidrug-resistant bacterial infections and detachable wound dressings [32]. In 2021, Liang Yuqing and his team developed a series of highly promising antioxidant and antibacterial self-healing hydrogels for the promotion of skin wound healing and treatment of methicillin-resistant Staphylococcus aureus (MRSA) infection-induced wounds, employing dual-dynamic-bond cross-linking among ferric iron (Fe), protocatechualdehyde (PA) containing catechol and aldehyde groups and quaternized chitosan (QCS) [33].

Therefore, it can be inferred that China holds a prominent position among the leading countries in the hydrogel biomedical application field. Xi’an Jiaotong University serves as a pivotal research institution, and Professor Guo Baolin and his students are highly esteemed scholars with significant citation records. To further explore additional applications of hydrogels in biomedical fields, researchers may consider collaborating with Professor Guo’s group. The extensive publication output and high citation rates of Guo Baolin’s team primarily revolve around novel hydrogel dressings development, indicating substantial contributions to this area and underscoring its potential for ongoing investigation. Among the 4 major applications of hydrogels in biomedical fields, the facilitation of wound healing has emerged as a prominent area of investigation. The combination of diverse drugs with hydrogels and the investigation of their role in wound healing for different types of wounds represent current research focal points. In future studies, researchers can explore the synergistic effects between various drugs and diverse hydrogels to further elucidate their potential in promoting wound repair.

### Research frontiers detection by keywords bursting analysis

We utilized the CiteSpace software to select the Top 25 exploding keywords, the details are shown in Fig. 19. According to the table, we can find that the keywords: “inflammation”, “injury” and “antibacterial” are both concerned with the wound healing field. The bursting of these keywords has been persisting until 2024, signifying that the advancement of innovative hydrogel dressings for wound healing remains at the forefront of scientific investigation. This finding substantiates the prevailing interest in utilizing hydrogels for wound healing applications, corroborating our co-citation analysis that underscores the substantial potential of hydrogels in promoting wound healing, while also exhibiting antibacterial and anti-inflammatory properties.

With the development of hydrogels in tissue engineering, regeneration medicine, drug delivery & controlled release, antibacterial material, wound healing, and other fields, there is an urgent need for researchers to identify novel research frontiers. Hence, it becomes imperative to employ bibliometric analysis techniques to discern current research trends and forecast future hotspots. It is worth noting that the bursting keywords “antioxidant” and “oxidative stress” prove that the researchers have discovered new applications for hydrogels - dealing with oxidative stress.

A physiological condition known as oxidative stress arises when excessive intracellular chemicals with oxidative characteristics upset the intracellular REDOX equilibrium. Oxidative stress causes cell damage, tissue aging, and the emergence of several illnesses because it causes biological molecules like proteins, lipids, and DNA to be destroyed by free radicals and other oxidants within the cell [34]. Two significant molecules that are pivotal in the oxidative stress process are reactive oxygen species (ROS) and reactive nitrogen species (RNS). ROS include free radicals and superoxide radicals (O_2_•^−^), hydrogen peroxide (H_2_O_2_), hydroxyl radical (•OH), and so on. These molecules are very oxidizing because they have unpaired electrons [35]. Overly high ROS levels can result in oxidative stress, which in extreme circumstances can cause serious cell damage and illness. Nitrogen peroxide and nitric oxide are components of RNS. Reactive nitrogen overproduction also results in oxidative stress, which harms cells and biomacromolecules [36]. As a result, oxidative stress may be reduced and the development of several illnesses can be avoided by efficiently eliminating excess ROS and RNS from organisms. Early in 2011, Victor W. Wong and his colleagues created pullulan hydrogels - a new polymer with strong antioxidant capabilities to fight oxidative stress. Research has indicated that the use of hydrogel based on pullulan considerably improves the mesenchymal stem cells’ ability to migrate and survive in conditions of severe oxidative stress, which in turn aids in the healing and repair of wounds. When subjected to high levels of oxidative stress, the pullulan-based hydrogel functions as a “sacrificial substrate” by interacting with free radicals, so protecting stem cells. By interacting with ROS, the hydrogel lowers the quantity of free radicals in living things and lessens the harm that oxidative stress causes to stem cells [37]. However, from 2012 ~ 2019, the use of hydrogels to combat oxidative stress didn’t become a hot topic for scientific inquiry. However, there has been a noticeable increase in the quantity of oxidative stress-related terms since 2020, indicating that researchers are growing increasingly interested in anti-oxidative stress strategies that were previously ignored. This pattern suggests with confidence that the application of hydrogels in the fight against oxidative stress is still uncharted territory that needs more study.

## Summary and prospects

In this study, we thoroughly examined a large number of publications published between 1973 and the present about the application of hydrogels in biological fields. We conducted a quantitative and visual study to highlight the findings and developments in this field using CiteSpace analytic software. First, we conducted a quantitative analysis of several factors, including authors, institutions, nations, journals, and yearly publishing output. Overall, hydrogel biomedical applications represent an expanding field that has witnessed rapid development in recent years with a progressively increasing number of publications annually. Our quantitative analysis revealed the majority of top-ranked publications based on a total number of publications, publishing institutions, and authors. Most of them originated from the USA and China, highlighting these two nations’ outstanding contributions to this domain. Through keyword analysis during each period studied, we identified key research areas and emerging directions for hydrogel biomedical applications. Finally, our focus was directed towards co-cited literature which demonstrated promising potential for utilizing hydrogels in promoting wound healing. Previous reviews on hydrogels have primarily focused on their application in specific diseases, lacking a comprehensive analysis of research trends in the biomedical field from a macro perspective and failing to provide adequate guidance for future researchers. Moreover, these reviews have typically selected a limited number of articles with time spans usually less than two decades, which does not fully encompass the entire historical progression of hydrogel research in biomedicine. In contrast, our study spans a broad temporal range by incorporating articles from the earliest publications up to the present day, effectively illustrating the evolutionary trajectory of hydrogels within the biomedical field. Additionally, it comprehensively examines various subsegments within biomedicine to identify prominent areas and offer valuable insights for novice researchers. Professionals will benefit from the information provided in this review as they work toward gaining a thorough understanding of the many applications of hydrogels in the biomedical industry. However, certain limitations are apparent in this study. In comparison to conventional reviews, CiteSpace analysis may lack thoroughness. For example, CiteSpace software is disabled to clearly distinguish between the first author and the corresponding author. Nevertheless, we firmly believe that ongoing efforts by the CiteSpace research team will continuously enhance this software and ultimately address these limitations while providing more precise analytical conclusions. It is anticipated that CiteSpace software-based comprehensive analyses on emerging knowledge domains can offer novel insights for researchers seeking emerging research trends.

Additionally, due to the limited length of this paper, some related fields, such as the crosslinking of hydrogels, could not be covered in detail. Crosslinking reactions involve bonding two or more molecules (typically linear molecules) together to form a more stable molecular network structure. This reaction transforms linear or mildly branched-chain macromolecules into a three-dimensional network structure, enhancing properties such as strength, heat resistance, wear resistance, and solvent resistance [38]. There are two main types of crosslinking methods: physical and chemical. Physical hydrogels are formed through weak interactions such as ionic interactions, hydrogen bonds, π-π packing, van der Waals forces, and hydrophobic interactions. Although they are easy to synthesize, physical hydrogels have limited control over mechanical properties and exhibit poor biocompatibility, thus their usage in the biomedical field is relatively low [39]. Conversely, chemical hydrogels possess superior mechanical properties along with stability and biocompatibility, making them suitable for long-lasting tissue engineering applications [40]. The diverse effects of crosslinking in hydrogels warrant further exploration through bibliometric analysis in our future studies.

## Data Availability

No datasets were generated or analysed during the current study.
